# Co-expression of non-signaling CARs enhances EGFRvIII-specific CAR T-cell cytotoxicity against head and neck squamous cell carcinoma

**DOI:** 10.1186/s12885-026-17058-z

**Published:** 2026-09-29

**Authors:** Kathleen Grueter, Nicole Sander, Luisa Coen, Saskia Hüsken, Eileen Kleinfelder, Corinna Haist, Franziska Blaeschke, Kathrin Scheckenbach, Cornelia Monzel, Helmut Hanenberg, Constanze Wiek

**Affiliations:** 1 Department of Otorhinolaryngology, University Hospital Düsseldorf, Medical Faculty of Heinrich-Heine University, Düsseldorf, Germany; 2 Hopp Children’s Cancer Center Heidelberg (KiTZ), Heidelberg, Germany; 3 German Cancer Research Center (DKFZ), Heidelberg, Germany; 4 Faculty of Biosciences, Heidelberg University, Heidelberg, Germany; 5 Department of Experimental Medical Physics, Heinrich-Heine University, Düsseldorf, Germany; 6 2nd Institute of Physics, University of Stuttgart, Stuttgart, Germany; 7 Department of Pediatrics III, University Children’s Hospital Essen, University Duisburg-Essen, Essen, Germany; 8 Department of Pediatric Oncology, Hematology and Immunology, Heidelberg University Hospital, Heidelberg, Germany

**Keywords:** HNSCC, EGFRvIII, CAR T-cell therapy, Non-signaling CAR, Tumor-specific antigen (TSA), Immunotherapy, Antigen density, Dual-epitope engagement

## Abstract

**Background:**

Head and neck squamous cell carcinoma (HNSCC) remains difficult to treat due to heterogeneous antigen expression and an immunosuppressive tumor microenvironment. The epidermal growth factor receptor variant III (EGFRvIII) is a tumor-specific splice form with no detectable protein expression in normal tissues. Nevertheless, its low and often heterogeneous expression in HNSCC has limited its utility as target antigen for immunological therapies using chimeric antigen receptor (CAR) T-cells. In contrast, full-length EGFR is frequently overexpressed in HNSCC, but its expression in normal tissues prevents its use as a directly activating CAR target. Thus, we investigated here whether EGFR can instead provide additional target antigen engagement as a non-signaling CAR (nsCAR) for EGFRvIII CARs. The nsCAR was derived from Cetuximab, an EGFR-binding monoclonal antibody whose epitope is retained in EGFRvIII and was co-expressed with different EGFRvIII-specific signaling CAR to investigate whether additional receptor engagement could enhance target-cell recognition and cytotoxicity.

**Methods:**

Primary human T-cells were engineered to express EGFRvIII-specific CARs alone or in combination with EGFR-directed nsCARs. Functional activity was evaluated using in vitro cytotoxicity assays, time-resolved killing dynamics, and fluorescence microscopy to assess immunological synapse (IS) formation in HNSCC cell lines with defined levels of EGFRvIII expression.

**Results:**

EGFRvIII CAR T-cells exhibited clear target antigen density–dependent cytotoxicity. Co-expression of the EGFR nsCAR specifically enhanced tumor cell killing in selected CAR and target antigen combinations, with the strongest effects observed under conditions of limited baseline CAR activity, whereas the nsCAR alone was non-cytotoxic. Fluorescence imaging revealed co-localization of CAR and nsCAR molecules at the T-cell–target-cell contact interface.

**Conclusions:**

EGFR nsCAR co-expression can provide a modular strategy to enhance EGFRvIII CAR T-cell cytotoxicity under conditions of limited target antigen availability. These findings provide an in vitro proof of concept for additional target engagement through a non-signaling receptor and warrant further evaluation in more complex preclinical models.

**Supplementary Information:**

The online version contains supplementary material available at https://doi.org/10.1186/s12885-026-17058-z.

## Introduction

Head and neck squamous cell carcinoma (HNSCC) is the seventh most common cancer worldwide with 890,000 cases diagnosed and 450,000 deaths per year [1, 2]. Treatment modalities for HNSCC primarily comprise combinations of surgery, radiation therapy, chemotherapy, and targeted therapies, including antibody therapies with the epidermal growth factor receptor (EGFR)-antibody Cetuximab and immune checkpoint inhibitors (ICIs) such as Nivolumab or Pembrolizumab, targeting the immunosuppressive PD-1/PD-L1 interaction between the cytotoxic T-lymphocytes and the malignant cells within the tumor microenvironment (TME) [3–5]. Still, the 5-year overall survival of HNSCC remains poor, as up to half of patients with locally advanced disease develop locoregional recurrence and/or distant metastases [3, 4].

Targeted personalized therapies with chimeric antigen receptors (CARs) represent a new powerful immunotherapeutic strategy to redirect autologous T-cells against antigens expressed on malignant or non-malignant cells. When expressed on T-cells, CARs enable efficient recognition and elimination of antigen-positive cells in an HLA-independent manner [6]. While CAR T-cell therapy has achieved major clinical breakthroughs in hematological malignancies of the B-cell lineage, its translation to solid tumors remains limited due to substantial challenges (reviewed in [7]), including (i) heterogeneity of target antigen expression among malignant cells; (ii) restricted trafficking and infiltration of CAR T-cells into tumors, which comprise a complex milieu of malignant cells, stromal fibroblasts, extracellular matrix, vasculature, and immunosuppressive cells; (iii) a hostile tumor microenvironment characterized by hypoxia, nutrient deprivation, and elevated immunosuppressive mediators; and (iv) limited persistence and functional fitness of CAR T-cells within tumor tissue. Nevertheless, some solid tumors have shown responsiveness to CAR T-cell therapy, particularly when a tumor-associated and relatively homogeneously expressed target antigen is available. Notable recent examples are the disialoganglioside GD2 in neuroblastoma or glypican-3 in several tumor entities, where recent clinical studies have demonstrated feasibility and specific activity of target antigen-directed CAR T-cells [8, 9]. Building on this concept, other tumor-specific antigens (TSAs) are being investigated to enable more selective targeting approaches in solid tumors [10].

One of the most frequently altered signaling pathways in HNSCC involves the epidermal growth factor receptor (EGFR), a transmembrane receptor tyrosine kinase that plays a crucial role in regulating cell proliferation, survival, and differentiation in epithelial tissues [11]. Overexpression or aberrant activation of EGFR has been detected in the majority of HNSCC cases and contributes to tumor progression and therapy resistance [12]. Among EGFR variants, the mutant form EGFRvIII is of particular interest because it is tumor-specific and lacks detectable protein expression in normal tissues. EGFRvIII arises from an *in-frame* deletion of exons 2 to 7 in the extracellular domain of EGFR, removing 267 amino acids and resulting in a shortened protein that is capable of constitutive, ligand-independent signaling [13–16]. Although the kinase activity of EGFRvIII is weaker than the enzyme activity of ligand-activated full-length EGFR, its residual constitutive activity is nevertheless sufficient to confer a survival advantage to tumor cells of various origins [17, 18]. Specifically, expression of EGFRvIII promotes cell proliferation, invasion, and tumor progression in different model systems and can indirectly enhance neoangiogenesis through upregulation of pro-angiogenic factors such as VEGF via downstream signaling pathways [19–21].

Importantly, the deletion generates a novel tumor-specific junctional epitope that is absent in full-length EGFR, thereby enabling selective targeting. This unique neoepitope, together with the restricted expression of EGFRvIII in tumor tissue, has made it an attractive candidate for immunotherapeutic strategies. Indeed, EGFRvIII CAR T-cells have demonstrated selective recognition of EGFRvIII-expressing tumor cells and have shown safety and feasibility in early-phase clinical trials in patients with recurrent glioblastoma (GBM); however, clinical efficacy has been limited, potentially due to antigen heterogeneity and antigen loss under therapeutic pressure [22, 23]. More recently, dual-targeting strategies have been explored to overcome these limitations. In a small cohort of patients with recurrent GBM, treatment with EGFRvIII CAR T-cells engineered to secrete a bispecific T-cell engager (BiTE) targeting EGFR and CD3ε resulted in encouraging initial responses and manageable toxicity following a single intraventricular administration. However, despite these promising early outcomes, disease progression ultimately occurred, likely reflecting the ongoing challenges of antigen escape and tumor heterogeneity in this setting [24, 25].

CAR T-cells are highly sensitive to target antigen density on tumor cells [26–28]. Their function can diminish considerably when target antigen expression levels fall below a critical threshold which varies depending on the specific target and the affinity and avidity of the single chain variable fragment (scFv) in the CAR construct [26, 29–31]. In addition, considering the tumor heterogeneity and the specific down-regulation or loss of target antigen expression, different strategies have been proposed to improve tumor recognition, including the co-expression of a second CAR in bicistronic constructs or the use of tandem CAR designs targeting multiple tumor-associated antigens [32–35]. However, the functional consequences of simultaneously engaging two distinct antigens – particularly with respect to CAR T-cell activation, persistence, and susceptibility to antigen-loss variants – are complex and remain poorly understood.

Here, we explored a modular receptor approach based on the co-expression of a fully functional 2nd generation CAR with CD28 costimulation, specifically recognizing EGFRvIII, alongside a CAR without any signaling domains (non-signaling CAR, nsCAR) to provide additional target engagement without directly triggering T-cell activation. In the experimental model established here, the cetuximab-derived EGFR nsCAR and the EGFRvIII-specific CAR can engage distinct epitopes retained on EGFRvIII. We therefore hypothesized that additional non-signaling receptor engagement could enhance CAR T-cell activity under conditions of limited target antigen availability.

## Methods

### Cell culture

HEK293T and DK-MG cells were obtained from the German Collection of Microorganisms and Cell Cultures (DSMZ, Germany). HNSCC cell lines were obtained from the University of Michigan (UM, USA), the University of Turku (UT, Finland), or from the University of Düsseldorf (UD, Germany). Primary human fibroblasts (Fibro) from a healthy donor were described previously [36]. Several EGFR knockout (KO) cell lines, UM-14C^KO^, UT-24A^KO^ and UM-10B^KO^, were previously generated in our laboratory by using lentiviral CRISPR/Cas9 technology [37]. All adherent cells were cultured in DMEM GlutaMAX medium supplemented with 10% fetal bovine serum (FBS), 100 U/mL penicillin, and 100 µg/mL streptomycin (all from Thermo Fisher Scientific, Germany).

Primary human T-cells were collected from peripheral blood of healthy adult donors who gave informed consent according to the protocols approved by the local ethics committee of the University Hospital Düsseldorf. Peripheral blood mononuclear cells (PBMCs) were separated by density-gradient centrifugation using Ficoll-Paque (Cytiva Europe, Germany) and cultured in IMDM (Sigma-Aldrich, Germany) supplemented with 10% FBS, 100 U/mL penicillin, 100 µg/mL streptomycin, 2 mM L-glutamine (all from Thermo Fisher Scientific), and 100 U/mL interleukin-2 (IL-2; Proleukin; Novartis, Switzerland) on 6-well plates pre-coated with CD3 (Thermo Fisher Scientific) and CD28 antibodies (BD Biosciences, USA) to activate and expand T-cells, as described before [37]. All cells were maintained at 5% CO_2_, 95% humidity, and 37 °C.

### RT-PCR analysis for EGFRvIII identification

The endpoint RT-PCR was initially established as a qualitative screening method to identify cell lines with detectable EGFRvIII transcripts before a suitable flow-cytometric antibody for EGFRvIII surface detection became available. Total RNA was extracted from 5 × 10⁶ cells using the NucleoSpin^®^ RNA Kit (Macherey-Nagel, Germany) following manufacturer’s instructions. cDNA was synthesized via two-step reverse transcription-PCR. First-strand cDNA was generated from 1 µg total RNA using Random Hexamer primers (Thermo Fisher Scientific), and M-MuLV Reverse Transcriptase (New England Biolabs, USA). For *EGFRvIII* detection, a subsequent PCR was performed using gene-specific primers flanking the deletion of exons 2 to 7, as described by Sok et al. [38]: forward primer, 5′-ATGCGACCCTCCGGGACG-3′; reverse primer, 5′-ATTCCGTTACACACTTTGCGGC-3′. PCR amplification was performed using Phusion^®^ High-Fidelity DNA Polymerase (New England Biolabs). Following an initial denaturation at 98 °C for 30 s, 38 cycles were performed at 94 °C, 55 °C, and 72 °C for 1 min each for denaturation, annealing, and extension, respectively, followed by a final extension at 72 °C for 10 min. Amplification products were analyzed by agarose gel electrophoresis. For confirmatory cDNA sequencing, DNA fragments corresponding to *EGFR* and *EGFRvIII* were excised from the gel and subjected to sequencing by Seqlab-Microsynth (Germany).

### Lentiviral expression constructs

All lentiviral vectors are derivatives of the puc2CL6 vector [39] and were generated using standard cloning techniques. Constructs used for the comparative analysis of different promoters were generated by PCR amplification of the promoters from donor plasmids and subsequently cloned into our standard enhanced green fluorescent protein (GFP) vector (p2CL6EGwo). The myeloproliferative sarcoma virus (MPSV), human phosphoglycerate kinase 1 (hPGK), spleen focus-forming virus (SFFV), and human elongation factor 1-α (hEF1α) promoter constructs were also previously published [40–42]. Human promoters derived from the promoter regions of the *FANCC* and *FANCG genes (personal communication by Prof. Dr. Detlev Schindler*,* Institute of Human Genetics*,* University of Würzburg*) and a promoter from murine *phosphoglycerate kinase* (*mPGK*) gene were also included in the promoter analysis [43].

The EGFR cDNA was cloned out of pBABEpuro-ERBB2 (Addgene plasmid # 40987) [44] in our lentiviral vector and then the EGFRvIII splice form was generated by removing exons 2–7 in the EGFR gene via overlap PCR. EGFR protein sequence information corresponds to UniProt accession P00533. EGFRvIII corresponds to the natural EGFR variant VAR_066493 (deletion of amino acids 30–297). To express EGFRvIII and neomycin phosphotransferase *npt II* (NEO) genes separated by a ribosome entry site (IRES) at different levels, three promoters were chosen (MPSV, hEF1α and hPGK).

We generated CAR constructs directed to EGFRvIII based on scFvs derived from five mAbs: murine 3C10 (CAR 9 referred to CAR EGFRvIII^A^) and humanized murine 3C10 (CAR 6 referred to CAR EGFRvIII^B^; CAR 8 referred to CAR EGFRvIII^C^; CAR 1 referred to CAR EGFRvIII^D^) from Novartis patent US2017/0008963A1 [45] and humanized 139 scFv (referred to CAR EGFRvIII^E^) from patent WO2012/138475A1 [46]. All EGFRvIII scFv sequences (CAR EGFRvIII^A−E^) were codon optimized for human using the internal algorithm of Thermo Fisher GeneArt and cloned into our self-inactivating lentiviral vector expressing a CD19 CAR with CD8 leader and the CD34-derived c6 hinge off a viral MPSV U3 promoter [47]. All 2nd generation CARs were inserted behind tagBFP (referred to as BFP) or mCherry in front of the T2A site [41, 42]. The control FMC63-based CD19, cetuximab-based EGFR, DRB2-based CD33, and BIWA8-based CD44v6 CAR constructs were previously published [37, 42, 47, 48]. The FMC63-based CD19 and cetuximab-based EGFR scFvs were used for nsCAR design with the human Ig heavy chain leader peptide under control of the MPSV U3 promoter and equipped with a NGFR-derived hinge region (n3), CD8 transmembrane, and without costimulatory domains [49]. The codon-optimized firefly luciferase/GFP fusion (LucGFP) vector was previously published [47, 50].

### Lentiviral production and cell transduction

Lentiviral particle production in HEK293T cells was carried out as previously described [41, 42, 50]. For pseudotyping, either vesicular stomatitis virus glycoprotein (VSV-G; 6 µg per transfection) or codon-optimized baboon endogenous virus envelope lacking the R-peptide (BaEV-Rless; 1 µg per transfection) was used. Briefly, cell culture supernatants containing replication-deficient lentiviral particles were collected 48 h post-transfection, filtered through a 0.45-µm filter, and either used directly or concentrated 5-fold by centrifugation at 10,000 × g for 2 h at 4 °C. Viral supernatants were applied fresh or stored at − 80 °C until use. Transduction of HNSCC cell lines and CD3/CD28-prestimulated primary human T-cells was performed in the presence of 10 µg/mL protamine sulfate (Sigma-Aldrich). 72 h after expansion and transduction, CAR T-cells were enriched with the MACS system (magnetic cell sorting, Miltenyi Biotec, Germany) by CD34 microbeads or CD271/LNGFR MACSelect microbeads as described in Bister et al. [47, 49], following the manufacturer’s protocol.

### Flow cytometry analysis and sorting

To assess EGFR and EGFRvIII surface expression, HNSCC cells were stained with PE- or APC-conjugated EGFR antibodies (clone REA688; Miltenyi Biotec; PE: 130-110-528, APC: 130-110-539) and an EGFRvIII recombinant rabbit monoclonal antibody (clone RM419; Thermo Fisher Scientific; MA5-36216). The latter was detected using a PE-conjugated anti-rabbit IgG (H + L) F(ab’)_2_ fragment (Cell Signaling Technology, USA; 79408 S). All antibodies were used according to the manufacturer’s instructions. For the detection of CARs or nsCARs on T-cells in the three fractions (pre-MACS, flow-through, and post-MACS), staining was performed using FITC- or PE-conjugated CD34 antibodies (clone QBEND/10, Thermo Fisher Scientific; FITC: MA1-10204, PE: MA1-10205) and APC- or PE-Vio770-conjugated CD271 antibodies (clone ME20.4, Miltenyi Biotec; APC: 130-113-418, PE-Vio770: 130-133-422). After staining and washing, cells were resuspended in MACS buffer containing either PI (Thermo Scientific; P1304MP) or DAPI (Miltenyi Biotec; 130-111-570) for live/dead cell discrimination. Flow-cytometric analysis was performed by first identifying the main cell population based on FSC-A and SSC-A characteristics. Singlet cells were subsequently selected using FSC-A versus FSC-H, followed by exclusion of PI- or DAPI-positive non-viable cells. EGFR, EGFRvIII, CAR, and nsCAR expression was then quantified within the viable singlet-cell population. Unstained cells and appropriate negative antibody controls were used to define background fluorescence and expression gates. Data were acquired using either a MACSQuant X flow cytometer (Miltenyi Biotec) or CytoFLEX S flow cytometer (Beckman Coulter, USA), and analyzed using the FlowJo software (BD Biosciences).

Fluorescence-activated cell sorting was performed at the Core Facility Flow Cytometry at the Heinrich-Heine University Düsseldorf, Germany. For sorting of EGFRvIII-positive cells, 5 × 10^6^ cells were stained with a PE-conjugated EGFR antibody (clone REA688; Miltenyi Biotec; PE: 130-110-528), which recognizes an EGFR epitope retained in EGFRvIII. Sorted cells were washed again, resuspended in fresh culture medium and further cultured under standard conditions.

### Cytotoxicity assay

#### CellTiter96^®^ AQueous one solution cell proliferation assay

To assess the cytotoxicity of effector T-cells, the CellTiter96^®^ AQueous One Solution Cell Proliferation Assay (Promega, USA) was used according to the manufacturer’s recommendations. Briefly, target cells (2 × 10^4^ target cells per well) were seeded in tissue culture-treated 96-well plates and incubated overnight at 37 °C. On the following day, MACS-selected transduced T-cells were added at different effector to target cell (E: T) ratios ranging from 3:1 to 0.01:1. Co-cultures were incubated for 16–20 h at 37 °C, unless stated otherwise. After incubation, wells were washed three times with PBS to remove non-adherent T-cells and dead target cells. The CellTiter96^®^ reagent was added according to the manufacturer’s instructions. The number of viable, adherent target cells was quantified by measuring absorbance at 490 nm using a Sunrise™ microplate reader (TECAN, Switzerland).

#### Luciferase luminescence cytotoxicity assay

The cytotoxicity of CAR T-cells on target cell lines (UM-14C^KO^+EGFRvIII^high^ and DK-MG) stably expressing the firefly luciferase transgene was measured via luciferase cytotoxicity assay. Briefly, target cells (2 × 10^4^ target cells per well) were seeded in white TCT 96-well plates with a transparent bottom (Nunclon 96 Flat Bottom White Polystyrol LumiNunc FluoroNunc, Thermo Fisher Scientific) and cultured overnight at 37 °C. On the following day, MACS-selected transduced T-cells were added at different effector to target cell (E: T) ratios ranging from 3:1 to 0.01:1. Co-cultures were incubated for 16–20 h at 37 °C. After incubation, the medium from each well was removed, and 100 µL of cell culture medium supplemented with D-luciferin (OZ Biosciences, France) at a final concentration of 0.75 µg/mL was added. The number of viable tumor cells was determined by measuring the luminescence with the Infinite M200 PRO plate reader (TECAN).

For both cytotoxicity assays, the percent lysis was calculated as: 100% − [(signal of target cells incubated with T-cells / signal of target cells alone) × 100], using absorbance for the MTS-based assay and luminescence for the luciferase-based assay. Occasionally, small negative lysis values were obtained when the measured signal of a co-culture exceeded that of the corresponding target-only control. These values reflect experimental variation around the reference signal and were retained without adjusting the values to zero. The two assay formats were used independently to assess CAR T-cell-mediated target-cell killing and yielded comparable cytotoxicity profiles for the principal EGFRvIII CAR constructs.

### Microscopy

For visualization of the IS, 2 × 10^4^ UM-14C^KO^+EGFRvIII^high^ cells were seeded in 8-well glass-bottom chamber slides (Ibidi, Germany). On the following day, doubly transduced CAR/nsCAR T-cells were stained with a FITC-conjugated CD34 antibody (Thermo Fisher Scientific) to label the c6 hinge region of the CAR and PE-Vio770-conjugated NGFR antibody (Miltenyi Biotec) to label the n3 hinge region of the nsCAR, according to the manufacturer’s protocols. Stained CAR/nsCAR T-cells were subsequently co-cultured with the target cells and analyzed by fluorescence microscopy using an IX83 microscope system (Olympus, Japan) equipped with a 60× oil-immersion objective (NA 0.65–1.25) and CellSens Dimensions software (Olympus, version 1.18). Additional confocal imaging was performed using *Nikon ECLIPSE Ti2* microscope (Nikon, Japan), equipped with a 100× oil-immersion objective (NA 1.49) and NIS-Elements AR-SP software (Nikon). CAR and nsCAR signals were detected in the FITC and PE-Vio770 channels, respectively; BFP-positive T-cells appeared blue and UM-14C^KO^+EGFRvIII^high^ target cells were visualized in phase contrast (PH). Contrast settings (channels: PH, 377/447 nm, 470/525 nm, and 545/620 nm) were adjusted to optimize immunological synapse (IS) visualization. Images were analyzed using OMERO software (version 5.30.1).

### Statistical analysis

Statistical analyses were performed using GraphPad Prism (GraphPad Software, Boston, MA, USA). Data are presented as mean ± SEM unless stated otherwise. Biological replicates and the exact number of independent T-cell donors are specified in the corresponding figure legends. For experiments using primary T-cells, each biological replicate represents one independent healthy donor, and matched analyses were performed across conditions generated from the same donor. Technical replicates were averaged before statistical analysis.

Promoter-dependent differences in EGFRvIII surface expression were analyzed using one-way repeated-measures ANOVA followed by Tukey’s multiple-comparisons test. Cytotoxicity data across different effector-to-target (E: T) ratios were analyzed using two-way repeated-measures ANOVA with donor as the matched experimental unit. Dunnett’s multiple-comparisons test was used when several CAR constructs were compared with a common control. For CAR/nsCAR co-expression experiments, including the time-course analyses, prespecified comparisons between the corresponding CAR/CD19 nsCAR and CAR/EGFR nsCAR conditions were assessed across the tested E: T ratios using the main effect of CAR/nsCAR condition. For time-course experiments, these comparisons were performed separately at each time point. The Geisser–Greenhouse correction was applied where appropriate. Multiplicity-adjusted *p-*values are reported for post hoc multiple-comparisons tests. A *p*-value of < 0.05 was considered statistically significant.

## Results

### EGFRvIII expression profiles of HNSCC cell lines

**Fig. 1:**
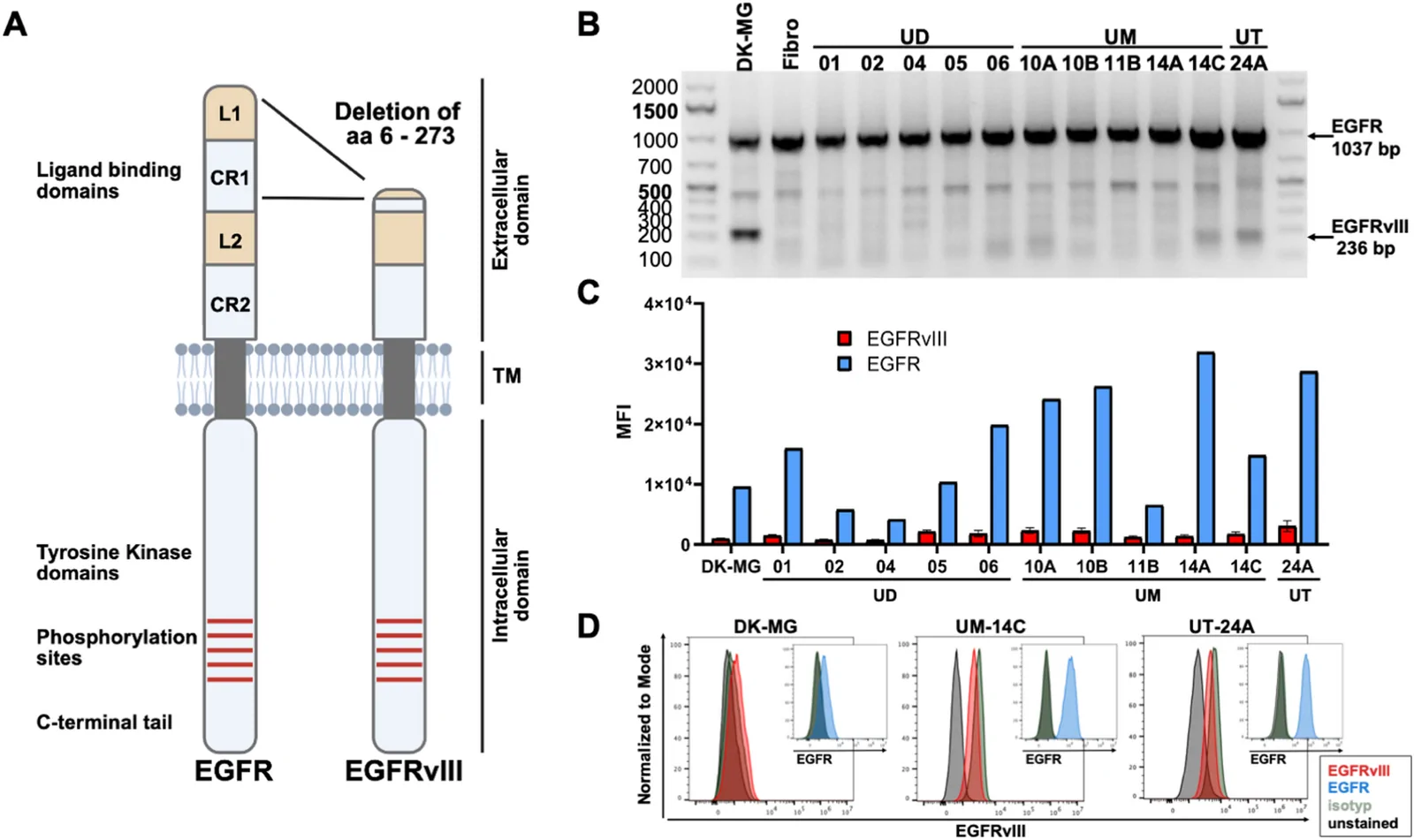
EGFRvIII expression in WT HNSCC cell lines. **A** Schematic structure of EGFR and EGFRvIII. The EGFRvIII variant is characterized by an in-frame deletion of exons 2–7 in the full-length EGFR gene. The domains of EGFR are shown, labeled domains L1 (also known as domain 1), CR1 (domain 2), L2 (domain 3) and CR2 (domain 4), respectively (adapted from Gan et al. 2009 [53]). **B** RT-PCR analysis of EGFRvIII mRNA expression. The presence of EGFRvIII mRNA was assessed in HNSCC cell lines by RT-PCR. A representative ethidium bromide-stained agarose gel shows PCR products for EGFR (1037 bp) and EGFRvIII (236 bp). Primary human fibroblasts (Fibro) served as negative control for EGFRvIII, while DK-MG cells were used as a positive control. Multiple HNSCC cell lines were analyzed. **C** and **D** Flow cytometric analysis of EGFRvIII protein expression. Cell lines were stained using a recombinant rabbit monoclonal EGFRvIII antibody followed by a PE-conjugated anti-rabbit IgG (H + L) F(ab’)₂ secondary antibody. EGFR expression was detected using APC- or PE-conjugated human EGFR antibodies. **C** Relative mean fluorescence intensity (MFI; y-axis) of EGFRvIII and EGFR expression in HNSCC cell lines. EGFRvIII data represent the mean ± SEM of three independent experiments. **D** Representative histograms of EGFRvIII (red), EGFR (blue), isotype staining with secondary antibody or isotype alone (dark green), and unstained cells (black). Normalized cell count is plotted against the fluorescence intensity

To qualitatively assess EGFRvIII mRNA expression in HNSCC, a panel of established HNSCC cell lines was analyzed by RT-PCR using primers flanking the deletion of exons 2 to 7 (Fig. 1A). This primer design allowed discrimination between the truncated EGFRvIII (236 bp) and the full-length EGFR transcript (1037 bp) [38]. As shown in Fig. 1B, full-length EGFR mRNA (1037 bp) was detected in all HNSCC cell lines, in primary human fibroblasts (Fibro), and in the glioblastoma line DK-MG which has been reported to be EGFRvIII-positive [51]. In addition to DK-MG cells as positive control, clear EGFRvIII PCR products were only observed in the HNSCC cell lines UM-14C and UT-24A. The specificity of all PCR products was confirmed by sequencing. On protein level, flow cytometric analysis (Figs. 1C–D) demonstrated cell-surface expression of EGFR (blue bars) across all HNSCC cell lines, albeit with variable intensities. In contrast, when using a previously described monoclonal antibody against the EGFRvIII-specific epitope generated by the in-frame deletion of exons 2–7 [52], EGFRvIII-specific surface expression (red bars) was not reliably detected on any of the analyzed cell lines under the conditions used, including the HNSCC cell lines and the DK-MG cells that were positive for EGFRvIII by RT-PCR. Thus, these results indicated that EGFRvIII protein levels on all cells analyzed were below the reliable detection limits for antibody staining using flow cytometry as read-out and did not demonstrate that EGFRvIII protein was completely absent.

### Generation of HNSCC cell lines with differential EGFRvIII expression

To reliably test EGFRvIII CAR T-cells, we sought to generate HNSCC cell lines lacking the full-length EGFR and overexpressing EGFRvIII at defined levels. For this purpose, we cloned seven internal promoter elements into a self-inactivating (SIN) lentiviral vector expressing the green fluorescent protein (GFP) in the previously described EGFR CRISPR/Cas9 knockout (KO) cells UM-14C^KO^ and UT-24A^KO^ [37] (Figure S1A). These included two viral U3 promoters derived from the spleen focus-forming virus (SFFV) and the myeloproliferative sarcoma virus (MPSV), four human promoters derived from the human *elongation factor-1 alpha* (*hEF1α*), *phosphoglycerate kinase* (*hPGK*), and the *Fanconi anemia C and G* (*FANCC* and *FANCG*) genes as well as one promoter derived from the murine *phosphoglycerate kinase* gene (mPGK) [40–43]. Transduction of EGFR KO cells at limiting dilutions with VSV-G-pseudotyped recombinant lentiviral particles enabled to directly compare the strength of the different promoters by measuring the transgene expression levels in flow cytometry. As shown in Figure S1B, the viral promoters SFFV and MPSV resulted in the strongest GFP expression levels at gene transfer efficiencies < 25% in both UM-14C^KO^ and UT-24A^KO^ cells. The lentiviral vectors with the mPGK, hPGK and hEF1α promoters achieved similar GFP expression levels that all were lower than those of the viral promoters. The expression levels of GFP in the cells transduced with the vectors containing the FANCC and FANCG promoters were very low. Based on these findings, SFFV was selected as promoter to achieve high EGFRvIII transgene expression levels, whereas the hEF1α and hPGK promoters were chosen with the intention to generate intermediate and low expression levels, respectively.

**Fig. 2:**
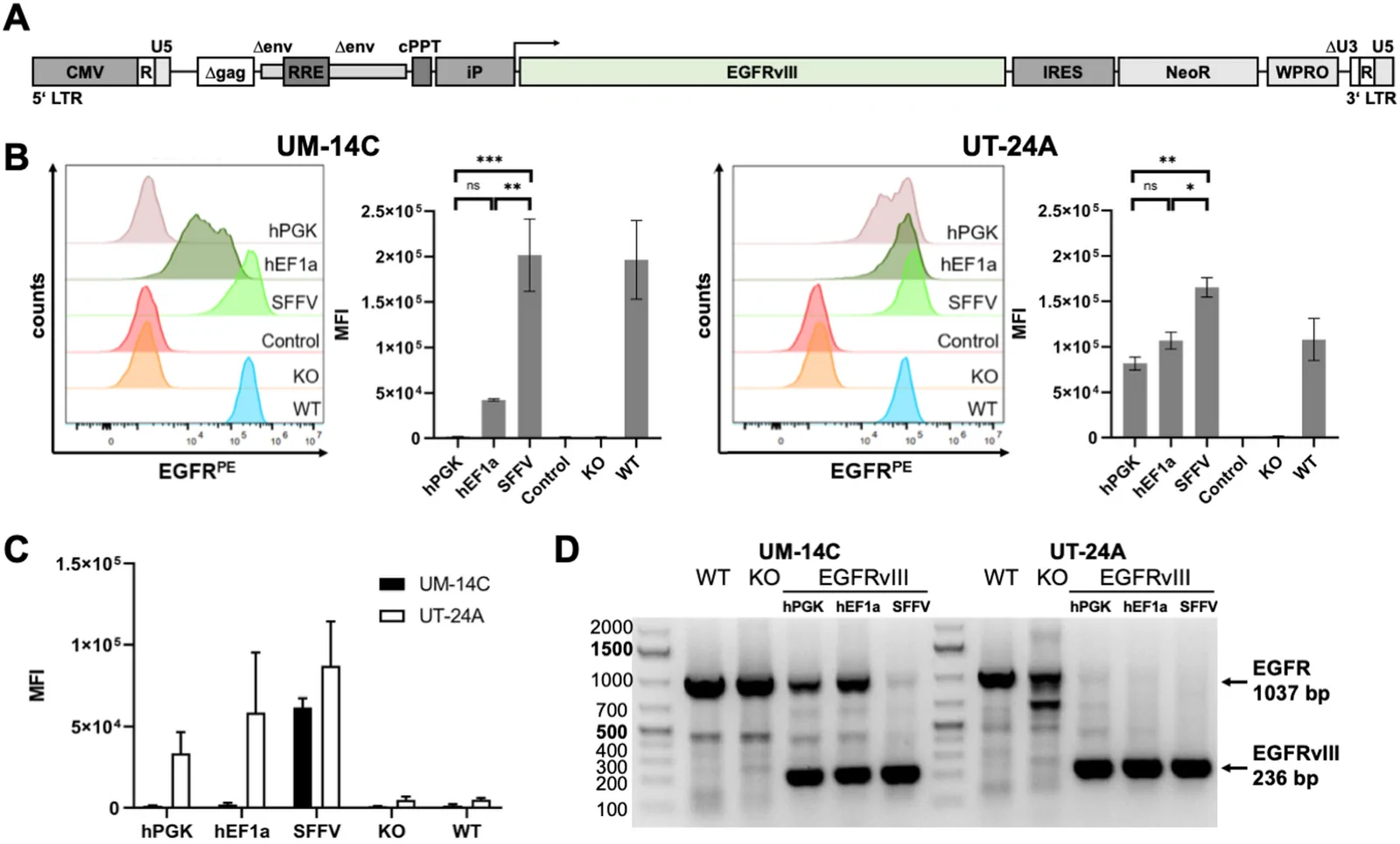
Artificial overexpression of EGFRvIII at different levels in HNSCC cell lines. **A** Schematic representation of the lentiviral EGFRvIII expression vector. The internal promoters (iP: hPGK, hEF1α and SFFV) drive expression of the EGFRvIII transgene and via an IRES site also the Geneticin resistance gene nptII (neoR). **B**-**D** UM-14C^KO^ and UT-24A^KO^ cells were transduced with LV EGFRvIII expression vectors. EGFRvIII-positive EGFR KO cells were further purified by flow sorting, generating new EGFRvIII-expressing cell lines. **B** and **C** Flow cytometric analysis of EGFRvIII surface expression after sorting. UM-14C and UT-24A cells were stained with (**B**) a PE-conjugated EGFR antibody for EGFR and EGFRvIII expression. Left: EGFR expression is shown by histogram. Right: Means ± SEM of MFI values of EGFRvIII-positive EGFR KO UM-14C and UT-24A cells as well as the corresponding UM-14C and UT-24A KO, WT and control cells transduced with an empty vector (data from three independent experiments) are shown. Differences between promoter groups within each cell line were analyzed using one-way repeated-measures ANOVA followed by Tukey’s multiple-comparisons test. p < 0.05 (*), p < 0.01 (**), p < 0.001 (***); ns, not significant. **C** EGFRvIII expression was additionally determined using an EGFRvIII-specific recombinant rabbit monoclonal antibody and a PE-conjugated anti-rabbit IgG (H + L) F(ab’)₂ secondary antibody. Data are presented as mean ± SEM from two independent experiments. **D** RT-PCR analysis of EGFRvIII mRNA expression. Presence of EGFRvIII mRNA was assessed in HNSCC cell lines artificially overexpressing EGFRvIII by RT-PCR. A representative ethidium bromide-stained agarose gel shows PCR products for EGFR (1037 bp) and EGFRvIII (236 bp). WT cell lines (UM-14C^WT^ and UT-24A^WT^), their corresponding EGFR KO derivatives, and the three EGFRvIII-overexpressing cell lines generated using different internal promoters were analyzed

We next generated EGFRvIII expression vectors with an IRES-neoR cassette incorporating these three internal promoters (Fig. 2A). VSV-G lentiviral particles were used to transduce UM-14C^KO^ and UT-24A^KO^ cells at limiting dilutions, followed by Geneticin^®^ selection until all non-transduced cells were eliminated. EGFRvIII expression was detected using a commercially available directly conjugated EGFR antibody targeting domain III of the extracellular region, which selectively recognizes EGFRvIII even in EGFR KO cells [54]. Importantly, the EGFR staining in WT cells detected both EGFR isoforms.

As shown in Fig. 2B, EGFR KO cells transduced with vectors containing the viral SFFV promoter exhibited high levels of EGFRvIII expression, whereas cells transduced with vectors containing the hEF1α promoter showed intermediate EGFRvIII protein expression levels by staining with an EGFRvIII-specific antibody (Fig. 2C). Importantly, EGFRvIII surface staining was readily detectable in both engineered cell populations, confirming that the antibody and flow-cytometric assay reliably detect EGFRvIII when expressed at elevated levels. Subsequent experiments were therefore performed with cells where the EGFRvIII cDNA was driven by either the SFFV or hEF1α promoters, hereafter referred to as EGFRvIII^high^ and EGFRvIII^mid^, respectively, to indicate their relative expression levels within the engineered cell-line model.

RT-PCR analysis with primers flanking the deletion of exons 2 to 7 was used to specifically confirm the presence of EGFRvIII transcripts in the engineered EGFRvIII overexpressing HNSCC cell lines (Fig. 2D). Interestingly, despite efficient EGFR knockout at protein level (assessed by flow cytometry), full-length EGFR transcripts remained detectable in both WT and KO cells, consistent with the potential presence of CRISPR/Cas9-induced mutant EGFR transcripts that do not result in stable protein expression.

### Characterization of EGFRvIII-specific CAR T-cells

**Fig. 3:**
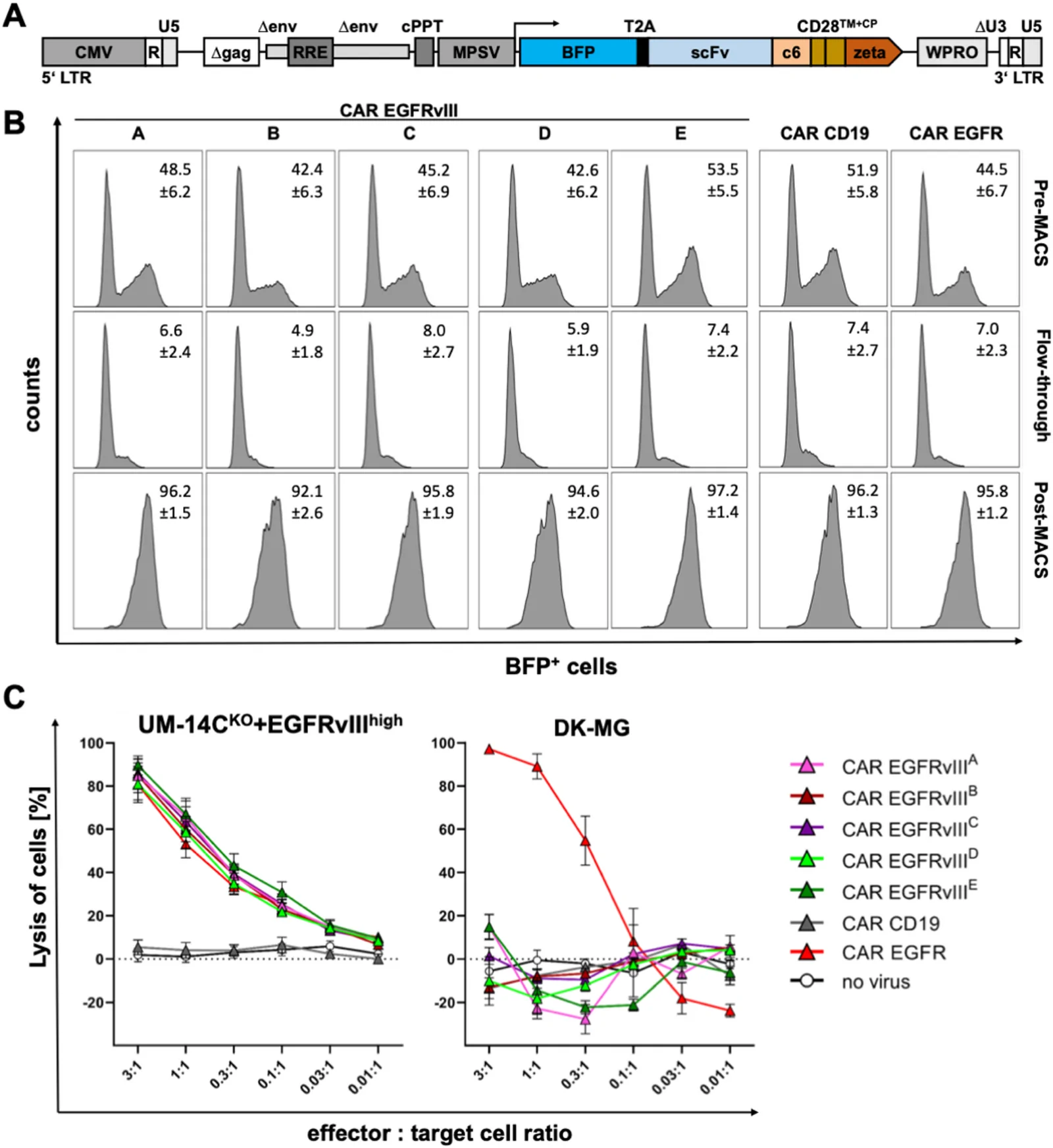
Design, enrichment, and cytotoxicity of CAR-positive T-cells. **A** Lentiviral CAR expression vector expressing the blue fluorescent protein TagBFP and a 2nd generation CAR construct. The vector contains either CD19, EGFR, or EGFRvIII^A–E^-specific scFvs, a CD34-derived hinge region (c6) followed by the CD28 transmembrane (TM) and cytoplasmic (CP) domains and the CD3ζ signaling chain. A BFP reporter gene is co-expressed via a T2A sequence. Transgene expression is driven by the MPSV promoter. **B** Transduction and enrichment efficiency of CAR-positive T-cells. Transduced T-cells were analyzed by flow cytometry before magnetic separation (Pre-MACS), in the flow-through fraction and after MACS selection (Post-MACS). CAR-positive cells were quantified based on BFP co-expression. Representative histograms show CAR expression (x-axis) plotted against normalized cell count (y-axis). CAR-positive cells are shown as mean ± SEM (*n* = 3). **C** CAR T-cell cytotoxicity. The target cell lines UM-14C^KO^+EGFRvIII^high^ and DK-MG were co-cultured with CD19, EGFR and EGFRvIII^A–E^ CAR T-cells or untransduced T-cells (no virus) for 16 to 20 h and target cell viability was assessed using a luciferase-based viability assay. Target cell lysis according to the E: T ratio (x-axis) is plotted against the percentage of lysed target cells (y-axis). Data represent mean ± SEM of three independent biological experiments using T-cells from independent healthy donors, with *n* = 5 for UM-14C^KO^+EGFRvIII^high^ and *n* = 3 for DK-MG. Statistical analysis was performed using two-way repeated-measures ANOVA across the tested E: T ratios, followed by Dunnett’s multiple-comparisons test versus the CD19 CAR control

To characterize different EGFRvIII-specific CAR T-cells, five scFv sequences targeting EGFRvIII were obtained from the literature, optimized for human codon-usage and cloned into our 2nd generation CAR lentiviral backbone, as shown in Fig. 3A [37]. EGFR-specific CAR T-cells derived from the monoclonal antibody Cetuximab served as positive control, while CD19 CAR T-cells were used as negative control [37]. The TagBFP fluorescent protein was included upstream of the T2A sequence as a reporter for genetically modified cells. Primary human T-cells were transduced with the respective CAR vectors and enriched for CAR expression using CD34 microbeads as published previously [47]. Analysis of BFP-expressing cells demonstrated that the percentage of CAR positive T-cells ranged from 42 to 54% before MACS enrichment and exceeded 92% thereafter (Fig. 3B). Only samples with > 90% CAR T-cells were used for cytotoxicity assays.

To assess functional differences between the EGFRvIII CAR constructs, cytotoxicity was evaluated using UM-14C^KO^+EGFRvIII^high^ and the EGFRvIII-expressing DK-MG cells (Fig. 3C). All EGFRvIII CAR T-cell variants efficiently lysed UM-14C^KO^+EGFRvIII^high^ cells and showed significantly increased cytotoxicity compared to the CD19 CAR control (*p* < 0.0001). None of the EGFRvIII CAR constructs mediated increased cytotoxicity against DK-MG cells, when compared to the CD19 CAR T-cell control. In contrast, the cetuximab-derived EGFR CAR efficiently lysed DK-MG cells and showed significantly increased cytotoxicity compared with the CD19 CAR control (*p* < 0.05), demonstrating that DK-MG cells were susceptible to CAR T-cell-mediated killing and that the endogenous EGFRvIII protein expression on DK-MG cells was below a level required for detectable EGFRvIII CAR T-cell activity. Based on these results, two CAR constructs, EGFRvIII^D^ and EGFRvIII^E^, were selected for further characterization.

### EGFRvIII CAR T-cells exhibit cytotoxicity dependent on EGFRvIII expression levels

To evaluate the cytotoxic potential of EGFRvIII-specific CAR T-cells, we performed co-culture killing assays using WT cells (UD-06, UD-05, UM-14C, UT-24A) or their EGFR KO counterparts (UM-14C^KO^, UT-24A^KO^).

**Fig. 4:**
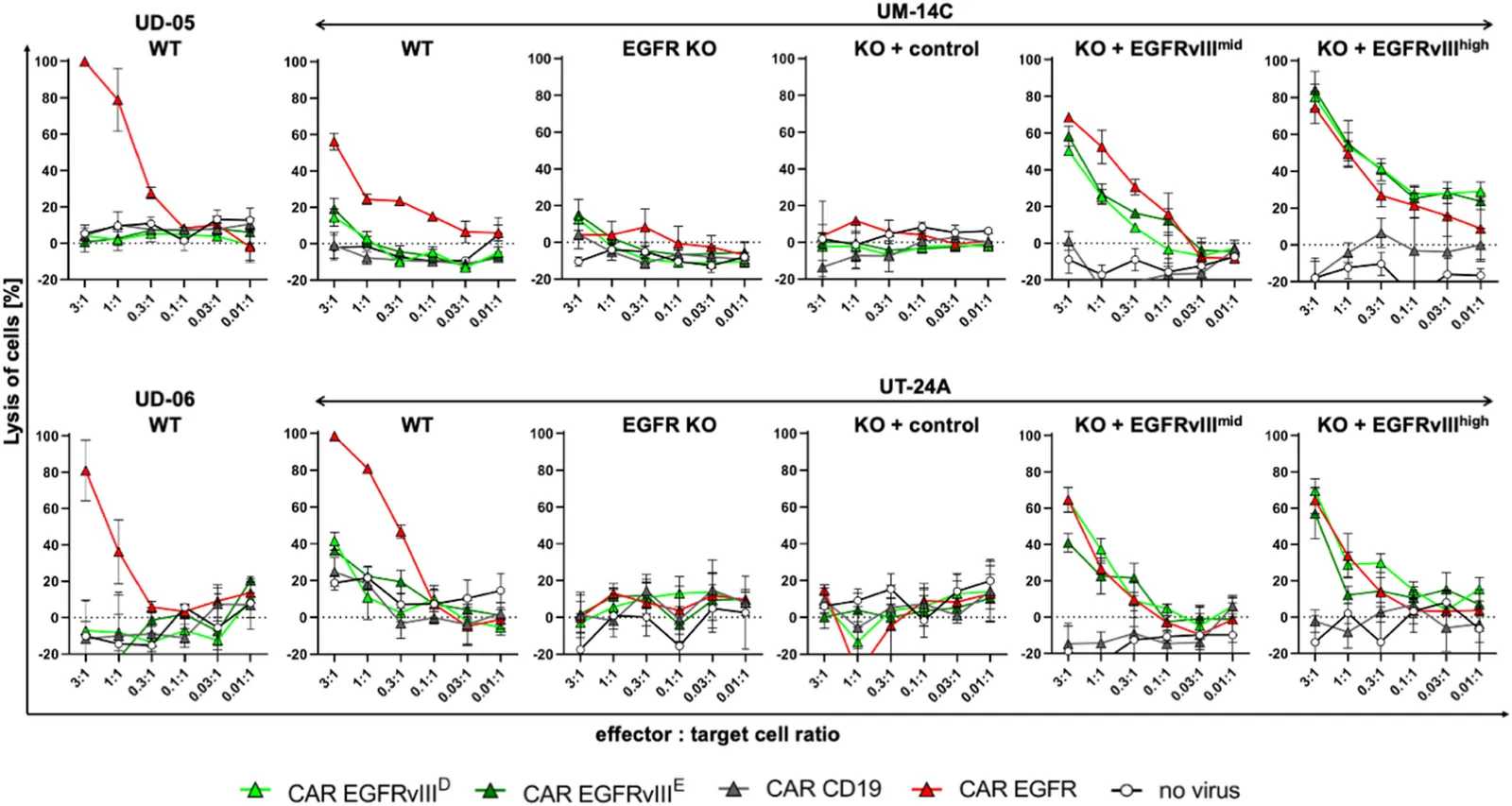
CAR T-cell cytotoxicity against different HNSCC cell lines. Different WT and genetically engineered UM-14C/UT-24A cells were co-cultured with CD19, EGFR, EGFRvIII^D^ or EGFRvIII^E^ CAR T-cells or untransduced (no virus) T-cells for 16 to 20 h and target cell viability was assessed using an MTS-based cytotoxicity assay. Target cell lysis at different E: T ratios (x-axis) is plotted against the percentage of lysed target cells (y-axis). Data represent mean ± SEM of three independent biological experiments using T-cells from three independent healthy donors (*n* = 3). Statistical analysis was performed using two-way repeated-measures ANOVA across the tested E: T ratios, followed by Dunnett’s multiple-comparisons test versus the CD19 CAR control

Additionally, we also used the latter two cell lines engineered to express intermediate or high levels of EGFRvIII for cytotoxicity assays. As shown in Fig. 4, EGFR CAR T-cells killed all four WT EGFR-positive cell lines (*p* < 0.05 for each cell line compared to CD19 CAR T-cells) but did not exert any detectable cytotoxic activity against the EGFR KO cells. Both EGFRvIII CAR T-cell variants showed limited cytotoxic activity against WT cells. However, the cytotoxic activity of EGFRvIII^D^ and EGFRvIII^E^ CAR T-cells against EGFRvIII-overexpressing UM-14C^KO^ cells increased with the EGFRvIII target antigen expression levels, with almost 60% lysis of UM-14C^KO^+EGFRvIII^mid^ cells and roughly 80% lysis of UM-14C^KO^+EGFRvIII^high^ cells at an effector-to-target ratio of 3:1, both significantly exceeding the cytotoxicity of the CD19 CAR control (*p* < 0.001 for all comparisons). For UT-24A cell models, the killing efficiency was generally lower and the differences in the cytotoxicity against UT-24A^KO^+EGFRvIII^mid^ and UT-24A^KO^+EGFRvIII^high^ cells were less pronounced (Fig. 4). Nevertheless, both EGFRvIII CAR variants showed significantly increased cytotoxicity compared with the CD19 CAR control at intermediate and high EGFRvIII expression levels (*p* < 0.05 for all comparisons). Notably, the cytotoxicity profiles of EGFRvIII^D^ and EGFRvIII^E^ CAR T-cells obtained using the MTS-based assay were comparable to those observed independently with the luciferase-based viability assay in UM-14C^KO^+EGFRvIII^high^ cells (Fig. 3C), demonstrating the concordance between the two cytotoxicity readouts.

### CARs and non-signaling CARs (nsCARs) can be co-expressed on the same T-cell

**Fig. 5:**
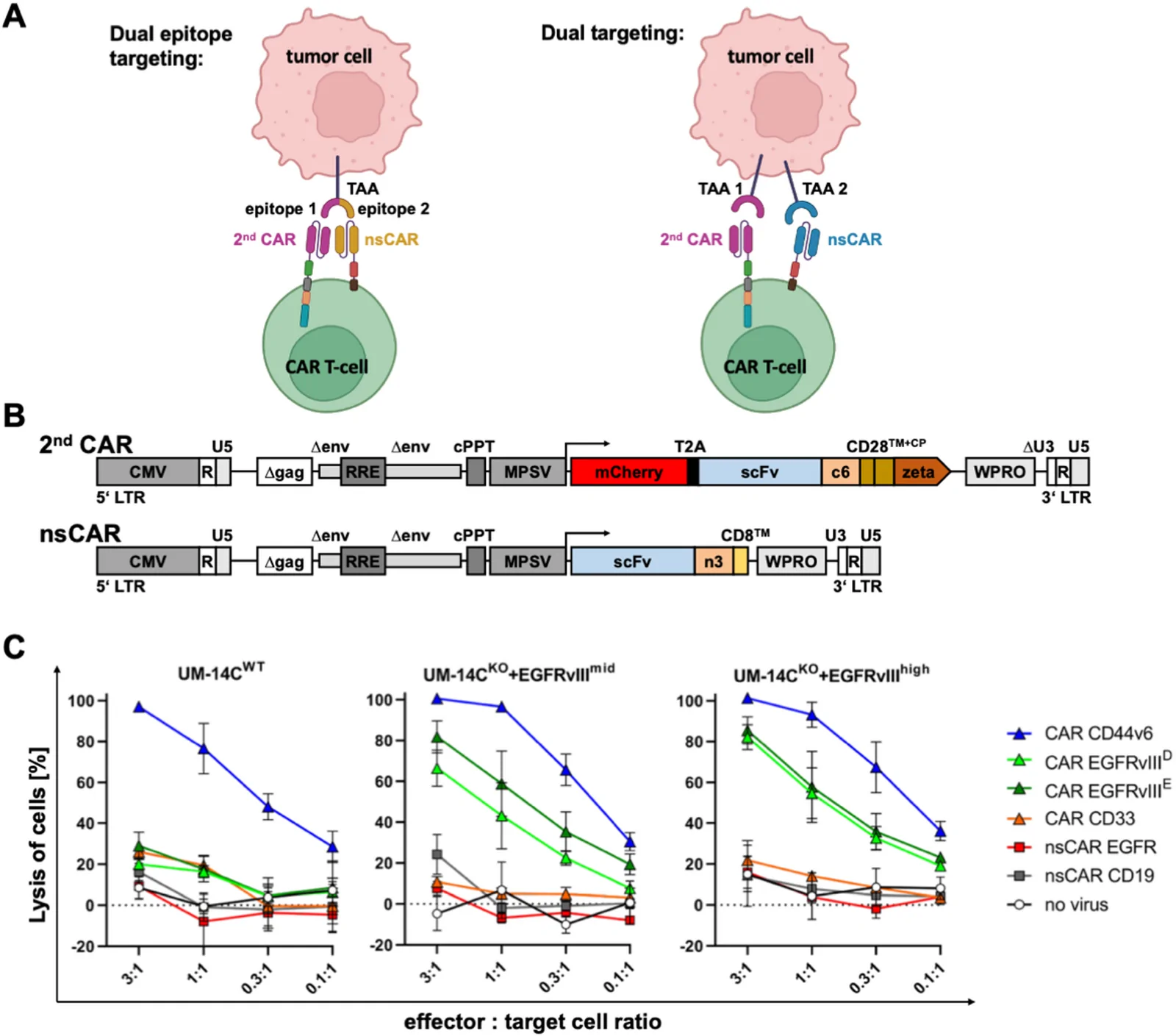
Concept, design and cytotoxicity of CAR and nsCAR T-cells. **A** Schematic representation of CAR/nsCAR co-expression strategies. 2nd generation CARs were combined with non-signaling CARs, which lack signaling domains and provide additional antigen/epitope engagement. **B** Lentiviral expression vectors encoding CARs or nsCARs. The 2nd generation CAR vector contains either CD44v6, CD33, or EGFRvIII^D−E^-specific scFvs, the c6-hinge, CD28 transmembrane and cytoplasmic regions, and the CD3ζ chain. An mCherry reporter was co-expressed via a T2A sequence. The nsCAR contains EGFR- or CD19-specific scFvs, the n3-hinge, and the CD8 transmembrane domain. **C** Killing efficiency of CAR and nsCAR T-cells against WT and genetically engineered UM-14C cells. Primary human T-cells were singly transduced with lentiviral constructs encoding CAR or nsCARs and CAR- or nsCAR-positive T-cells (> 90%) were co-cultured with UM-14C cells for 16 to 20 h and target cell viability was assessed using an MTS-based cytotoxicity assay. Target cell lysis at different E: T ratios (x-axis) is plotted against the percentage of lysed target cells (y-axis). Data represent mean ± SEM of three independent biological experiments using T-cells from three independent healthy donors (*n* = 3). Statistical analysis was performed using two-way repeated-measures ANOVA across the tested E: T ratios, followed by Dunnett’s multiple-comparisons test versus the CD33 CAR control

Unlike classical CARs, nsCARs consist only of an antigen-binding domain, a hinge, and a transmembrane region and therefore cannot induce T-cell activation or target-cell killing [55]. In order to enhance the modest cytotoxic activity of EGFRvIII CAR T-cells under conditions of limited target antigen availability, we decided to co-express the 2nd generation CARs and nsCARs on the same T-cell, thereby enabling dual-epitope recognition of the same target molecule or simultaneous targeting of two distinct antigens (Fig. 5A). To this end, we first generated mCherry-labeled CAR constructs targeting CD33 [47], EGFRvIII (D and E variants), and CD44v6 (a splice variant frequently expressed in HNSCC [42]). These signaling-competent CAR constructs were paired with either a CD19-directed nsCAR as a negative control or the EGFR-directed nsCAR as the experimental receptor (Fig. 5B). To minimize the risk of homologous recombination between the two CARs during lentiviral integration, we employed a leader sequence derived from the human Ig heavy chain (R) for the nsCARs – instead of the CD8 leader (J) used in the CAR vectors, and replaced the CD28 transmembrane domain with the counterpart of CD8. To enable flow cytometric detection and magnetic-activated cell sorting (MACS) of nsCAR-expressing cells, we incorporated an NGFR-derived hinge region (n3) instead of the CD34-derived c6 hinge [47, 49]. Standard cytotoxicity assays with single constructs showed that T-cells expressing the three signaling-competent CARs, namely CD44v6, EGFRvIII^D^ and EGFRvIII^E^, efficiently and specifically killed HNSCC target cells, with CD44v6 CAR T-cells showing significantly increased cytotoxicity across all three target-cell settings (*p* < 0.001) and EGFRvIII^D^ and EGFRvIII^E^ CAR T-cells specifically against EGFRvIII^mid^ and EGFRvIII^high^ cells (*p* < 0.05 for all comparisons), whereas the CD33 CAR control and nsCARs directed against EGFR and CD19 did not mediate detectable cytotoxicity (Fig. 5C).

To enable efficient co-expression of both constructs in the same T-cells, we generated lentiviral vectors pseudotyped with VSV-G for the CAR and BaEV-Rless for the nsCAR. After titration of the frozen supernatants, we co-transduced primary human T-cells with both vectors, thereby avoiding competition between the viral particles for the same cellular entry receptor and to facilitate efficient co-transduction [56]. As the shorter nsCARs consistently exhibited much higher transduction efficiencies (72–79%) than the CARs (6–19%) (Figure S2A), we enriched for CAR-positive T-cells using CD34 microbeads, yielding populations containing > 90% CAR-positive T-cells. Co-expression analysis demonstrated that up to 79% of T-cells co-expressed both CAR and nsCAR when EGFR nsCARs were used (Figure S2A). NGFR microbead-based enrichment was performed for cells transduced with nsCARs alone. Only MACS-enriched T-cells were used in subsequent functional analyses. Post-MACS cell viability was routinely assessed by flow cytometry and was generally > 90%. To assess whether nsCAR co-expression was associated with major differences in signaling-CAR expression after enrichment, we additionally compared CD34 staining in CAR-only and matched CAR/nsCAR T-cell products from a representative donor. Post-MACS signaling-CAR expression showed comparable mean fluorescence intensities between CAR-only T-cells and the corresponding T-cell products co-expressing either the EGFR or CD19 nsCAR across the CAR constructs used (Figure S2B).

### The cytotoxicity of EGFRvIII CAR T-cells can be enhanced by additional nsCARs

**Fig. 6:**
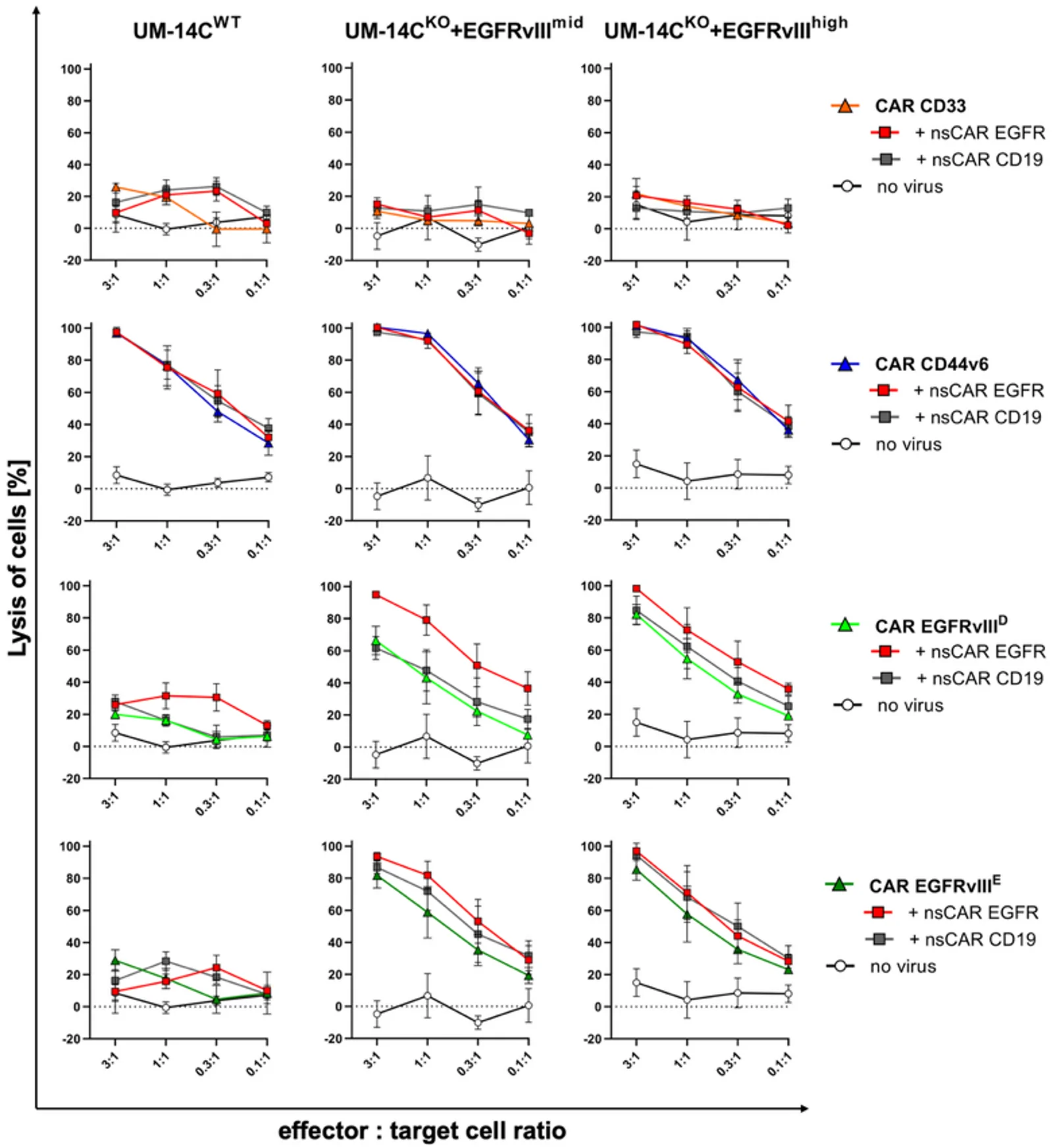
Killing efficiency of CAR/nsCAR T-cells. Primary human T-cells were singly or doubly transduced, and CAR-positive T-cells, including CAR/nsCAR double-positive cells, were enriched using CD34 microbeads. Cells were co-cultured with UM-14C cells for 16 to 20 h and target cell viability was assessed using an MTS-based cytotoxicity assay. Target cell lysis at different E: T ratios (x-axis) is plotted against the percentage of lysed target cells (y-axis). Data represent mean ± SEM of three independent biological experiments using T-cells from three independent healthy donors (*n* = 3). Statistical analyses were performed using two-way repeated-measures ANOVA across the tested E: T ratios, with donor as the matched experimental unit. Prespecified comparisons assessed the effect of EGFR nsCAR co-expression relative to the corresponding CD19 nsCAR control condition

Next, we co-cultured CAR/nsCAR co-expressing T-cells with UM-14C^WT^, UM-14C^KO^+EGFRvIII^mid^ and UM-14C^KO^+EGFRvIII^high^ target cells and assessed the cytolytic activity in standard killing assays. As shown in Fig. 6, no cytotoxicity was observed for CD33 CAR T-cells, irrespective of nsCAR co-expression. In contrast, CD44v6 CAR T-cells exhibited high cytotoxicity across all tested target-cell conditions, and EGFR nsCAR co-expression did not significantly alter cytotoxicity in any of these conditions. Cytotoxicity of EGFRvIII CAR T-cells against UM-14C^WT^ cells remained generally low. However, for EGFRvIII^D^ CAR T-cells, EGFR nsCAR co-expression resulted in a statistically significant increase in cytotoxicity compared with the corresponding CD19 nsCAR control (*p* < 0.01), whereas no significant difference was observed for EGFRvIII^E^ CAR T-cells. Against UM-14C^KO^+EGFRvIII^mid^ cells the limited cytotoxicity of EGFRvIII^D^ CAR T-cells was clearly enhanced by EGFR nsCAR co-expression, increasing the target-cell killing from ~ 65% to nearly 100% at an E: T ratio of 3:1, and from ~ 40% to ~ 80% at an E: T ratio of 1:1. Across the E: T ratios, this increase in cytotoxicity was significant for EGFRvIII^D^ CAR T-cells co-expressing the EGFR nsCAR compared with the corresponding CD19 nsCAR control (*p* < 0.05). In UM-14C^KO^+EGFRvIII^high^ cells, EGFRvIII^D^ CAR T-cells already exhibited high cytotoxicity, which was not significantly enhanced by EGFR nsCAR co-expression. A similar but not significant trend was also observed for EGFRvIII^E^ CAR T-cells when using UM-14C^KO^+EGFRvIII^mid^ cells as targets.

### Time-dependent effects of EGFR nsCAR co-expression on CAR T-cell cytotoxicity

**Fig. 7:**
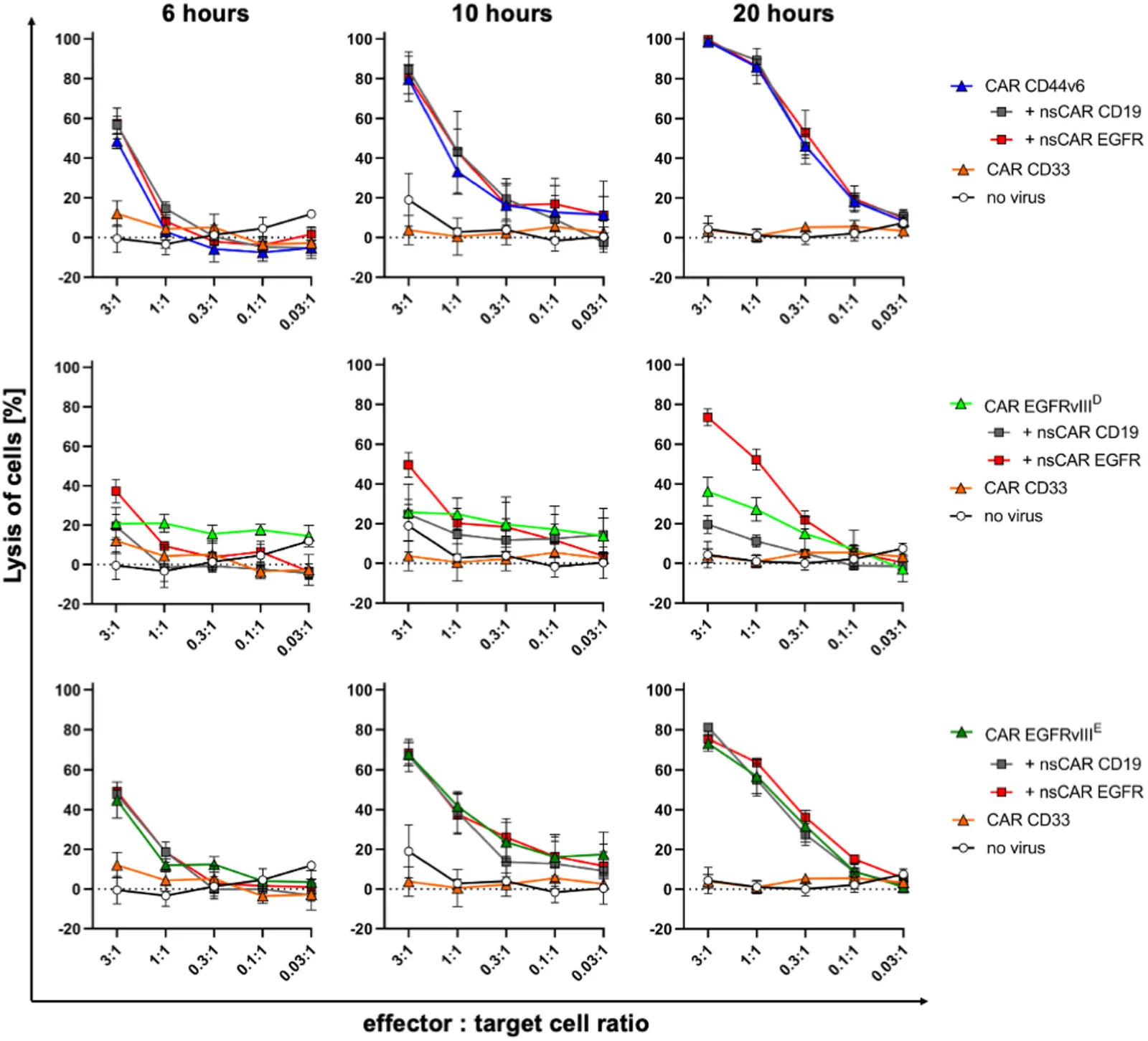
Killing dynamics of CAR/nsCAR T-cells. Primary human T-cells were transduced with an EGFRvIII^D/E^ or CD44v6-specific CAR alone or in combination with additional EGFR- or CD19-specific nsCAR constructs. CAR-positive T-cells, including CAR/nsCAR double-positive cells, were enriched using CD34 microbeads and co-cultured with UM-14C^KO^+EGFRvIII^mid^ cells for 6, 10, and 20 h and target cell viability was assessed using an MTS-based cytotoxicity assay. Target cell lysis at different E: T ratios (x-axis) is plotted against the percentage of lysed target cells (y-axis). Data represent mean ± SEM of three independent biological experiments using T-cells from three independent healthy donors (*n* = 3). Statistical analyses were performed separately for each time point using two-way repeated-measures ANOVA across the tested E: T ratios, with donor as the matched experimental unit. Prespecified comparisons assessed the effect of EGFR nsCAR co-expression relative to the corresponding CD19 nsCAR control condition

To understand the killing dynamics of CAR-only and CAR/nsCAR co-expressing T-cell products, MACS-enriched T-cells were co-cultured with UM-14C^KO^+EGFRvIII^mid^ cells and cytotoxicity was measured after 6, 10 and 20 h. As shown in Fig. 7, nsCAR co-expression did not significantly enhance the antitumor activity of EGFRvIII^E^ or CD44v6 CAR T-cells at any time point. In contrast, EGFRvIII^D^ CAR/EGFR nsCAR T-cells exhibited increased cytotoxicity at early time points compared with EGFRvIII^D^ CAR T-cells co-expressing the CD19 nsCAR control. Notably, at 10 h, EGFRvIII^D^ CAR/CD19 nsCAR T-cells lysed ~ 25% of target cells, whereas EGFRvIII^D^ CAR/EGFR nsCAR T-cells achieved ~ 50% lysis. However, these differences were not statistically significant. After 20 h, the effect of the EGFR nsCAR co-expression became more pronounced at an E: T ratio of 3:1: ~75% of target cells were lysed by EGFRvIII^D^ CAR/EGFR nsCAR T-cells, compared with ~ 20% by the corresponding CD19 nsCAR control. This increase in killing was statistically significant (*p* < 0.05 for EGFRvIII^D^ CAR/EGFR nsCAR T-cells compared to the corresponding CD19 nsCAR control).

### Differential recruitment of CAR and nsCAR at the immunological synapse (IS)

**Fig. 8:**
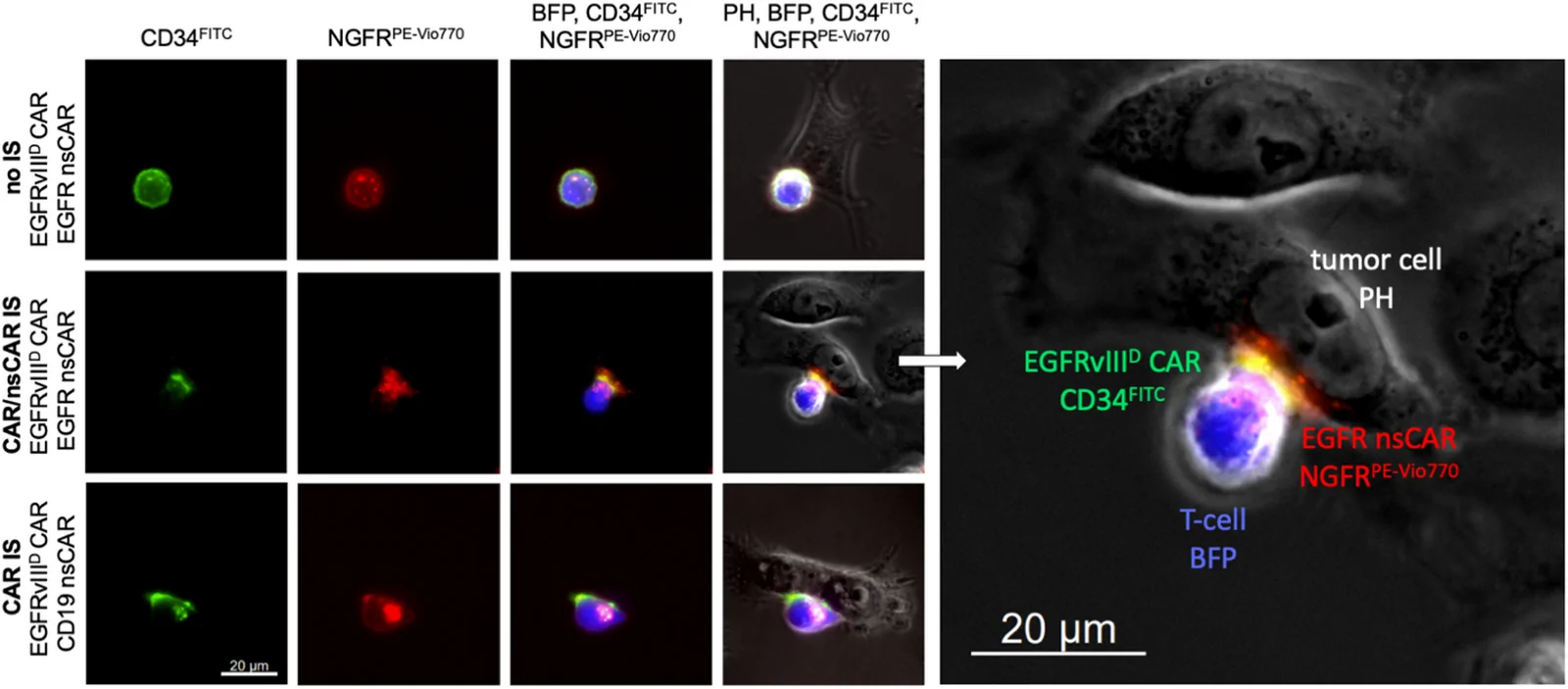
Visualization of the IS. The IS was analyzed by fluorescence microscopy, visualizing the EGFRvIII^D^ CAR (FITC-conjugated CD34 antibody, green) and EGFR or CD19 nsCAR (PE-Vio770-conjugated NGFR antibody, red) proteins on MACS-enriched CAR/nsCAR T-cells (BFP expression, blue) in relation to UM-14C^KO^+EGFRvIII^high^ cells (phase contrast). Contrast settings (channels: PH, 377/447 nm, 470/525 nm and 545/620 nm) were adjusted to optimize IS visualization

To investigate whether enhanced cytotoxicity of CAR/nsCAR T-cells can be linked to IS formation, we employed fluorescence microscopy to visualize the CAR and nsCAR protein distribution during the T-cell–tumor cell interactions. For these experiments, CAR/nsCAR T-cells were stained with hinge-specific antibodies (CD34^FITC^ for CAR, NGFR^PE−Vio770^ for nsCAR) and co-cultured with UM-14C^KO^+EGFRvIII^high^ cells. Additionally, CAR T-cells were identifiable by BFP co-expressed in the vector. Without target cell contact (Fig. 8, No IS), both CAR (green) and nsCAR (red) proteins were evenly distributed across the surface of CAR/nsCAR T-cells (blue). Upon target recognition (Fig. 8, CAR/nsCAR IS), both EGFRvIII^D^ CAR and EGFR nsCAR proteins accumulated at the IS, indicating coordinated recruitment of both constructs to the contact zone between the target and the effector cell. EGFRvIII^D^ CAR/CD19 nsCAR T-cells also formed synapses with the target cells (Fig. 8, CAR IS). However, in contrast to CAR proteins, which localized exclusively to the IS, the nsCAR proteins targeting CD19, which were not expressed on these tumor cells, did not localize to the IS. These results demonstrate that, in double-positive T-cells, CAR and nsCAR proteins are co-recruited to the IS when both target antigen epitopes are present on the tumor cell, consistent with simultaneous engagement of distinct epitopes on EGFRvIII.

## Discussion

CAR T-cell therapy has revolutionized the treatment landscape for hematologic malignancies of the B-cell lineage, yet its role in the therapy of solid tumors remains markedly limited due to the scarcity of tumor-specific antigens, heterogeneous target antigen expression profiles, the suppressive tumor microenvironment, restricted trafficking and infiltration of CAR T-cells into tumors and limited persistence and functional fitness of CAR T-cells [7, 57].

### EGFRvIII as a target antigen in HNSCC

As EGFRvIII is one of the few target antigens well established in glioblastoma with remarkable tumor specificity [15] and has also been described in HNSCC [38, 58, 59], the primary goal of the present study was to characterize the cytotoxic activity of five distinct EGFRvIII scFvs used as targeting moieties in 2nd generation CAR constructs on human T-cells. However, analysis of EGFRvIII expression in human HNSCC cell lines revealed that only two of eleven cell lines, UM-14C and UT-24A, were positive at the mRNA level by PCR. While endogenous EGFRvIII surface expression in these cell lines and in DK-MG cells could not be reliably detected by flow cytometry using a published commercial antibody [51], the same antibody produced clear signals in engineered EGFRvIII-overexpressing cells, demonstrating that the staining approach could detect EGFRvIII at higher expression levels. Detailed analysis of the HNSCC literature indicates that EGFRvIII expression frequencies were described as high as ~ 40% in early studies [38], whereas later analyses using larger cohorts and more stringent molecular detection methods (e.g., qRT-PCR, RNA-sequencing) have consistently reported EGFRvIII expression to be rare [60–62]. One study reported EGFRvIII protein expression in only 2 of 638 tumors (~ 0.3%) [62]. We therefore also included DK-MG cells, a glioblastoma cell line widely used as an EGFRvIII-positive reference model [51, 63], in our studies. However, cytotoxicity assays with the five EGFRvIII CAR T-cell variants against all three putatively EGFRvIII-positive cell lines (UM-14C, UT-24A and DK-MG) did not reveal any specific cytotoxicity, whereas all five EGFRvIII CAR constructs efficiently and specifically lysed engineered EGFRvIII-high target cells. Importantly, DK-MG cells were efficiently lysed by the cetuximab-derived EGFR CAR, demonstrating that this cell line is not intrinsically resistant to CAR T-cell-mediated killing. Together, these findings suggest that endogenous EGFRvIII, if present, is expressed at levels below the detection and/or functional activation thresholds of the respective assays. To systematically investigate the impact of antigen density on EGFRvIII CAR T-cell activation, we overexpressed the EGFRvIII cDNA in EGFR KO HNSCC cell lines, using lentiviral vectors with different internal promoters. Employing these engineered HNSCC cell lines with different EGFRvIII expression levels (EGFRvIII^mid^ and EGFRvIII^high^) as target cells allowed us to demonstrate that all five EGFRvIII-specific CAR constructs (EGFRvIII^A–E^) mediated robust cytotoxicity when EGFRvIII was present at sufficient levels and that all five EGFRvIII CAR constructs were specific, as they did not recognize either the parental EGFR KO or WT cells.

EGFRvIII has previously been explored as a target for immunotherapeutic approaches. Peptide-based vaccination strategies, particularly the EGFRvIII-specific vaccine rindopepimut (CDX-110), which targets the tumor-specific junctional epitope created by the exon 2–7 deletion, demonstrated robust immunogenicity with signs of clinical benefits in early-phase clinical trials in patients with glioblastoma [64]. However, the randomized phase III trial (ACT IV, NCT01480479) failed to improve overall survival of patients, with subsequent analyses suggesting that antigen loss and tumor heterogeneity may have contributed to the lack of clinical benefit [65]. After initial signs of activity in glioblastoma, EGFR-targeting antibody–drug conjugates, including depatuxizumab mafodotin (ABT-414), which also recognizes EGFRvIII-expressing tumors, failed in phase II/III clinical trials (NCT02343406, NCT01800695) due to lack of significant survival benefit and ocular toxicity [66, 67]. Preclinical studies demonstrated that EGFRvIII CAR T-cells can selectively recognize and eliminate EGFRvIII-expressing tumor cells [45, 68, 69], and a first-in-human phase I/II clinical trial in patients with recurrent GBM (NCT02209376) showed that intravenous infusion of autologous EGFRvIII CAR T-cells following tumor resection was safe and feasible, and demonstrated CAR T-cell trafficking to tumor sites [22]. However, no objective tumor responses were observed and tumor recurrence occurred in all cases, with findings suggesting that antigen heterogeneity and treatment-associated antigen loss may have contributed to the limited efficacy [22]. A second clinical trial of EGFRvIII CAR T-cells against recurrent GBM (NCT01454596) showed safety and transient efficacy but did not result in durable therapeutic responses, with findings indicating potential contributions of antigen escape and adaptive resistance mechanisms [23].

### Enhancing EGFRvIII CAR T-cell cytotoxicity under limited target antigen expression

Different strategies have been developed to enhance the killing efficiency of CAR T-cells in solid cancers [57, 70, 71]. Interestingly, Choi et al. have genetically modified EGFRvIII CAR T-cells to secrete BiTEs that recognize EGFR and CD3ε [25]. This strategy was later translated into the clinic using related T-cell–engaging antibody molecules (TEAMs), where a phase I/II study employing a single intraventricular administration in a small cohort of patients with recurrent glioblastoma resulted in encouraging initial responses and manageable toxicity. However, despite these promising early outcomes, disease progression ultimately occurred, likely reflecting the ongoing challenges of antigen escape and tumor heterogeneity in this setting [24]. As EGFR is widely expressed in HNSCC and can be used as target antigen for CAR T-cells [37], we initially designed a cetuximab-based non-signaling CAR (nsCAR) targeting domain III (L2) of EGFR as an accessory receptor for EGFRvIII CAR T-cells. However, as the endogenous expression of EGFRvIII in all HNSCC cell lines and also the DK-MG cells was too low to trigger the CAR signaling, the principal functional experiments in the present study were performed using EGFR-knockout cells engineered to overexpress EGFRvIII. In this model, full-length EGFR is absent, while EGFRvIII retains the epitope recognized by the cetuximab-derived receptor. The enhancement observed in our work therefore reflects an additional engagement of a second epitope on EGFRvIII rather than independent targeting of full-length EGFR and EGFRvIII. This dual-epitope engagement may increase functional avidity and thereby facilitate CAR T-cell activity under conditions of limited target antigen availability.

Co-expression of the EGFR nsCAR did not enhance the cytotoxic activity of EGFRvIII^E^ CAR T-cells, as this CAR construct alone already mediated highly efficient killing of both intermediate- and high-EGFRvIII-expressing cells, comparable to the cytotoxic activity of the control CD44v6 CAR T-cells. In contrast, when combined with the EGFR nsCAR, EGFRvIII^D^ CAR T-cells exhibited markedly improved cytotoxicity in our standard overnight assays, with maximal killing increasing from approximately 65% to near complete target clearance at the 3:1 effector-to-target ratio. Compared with EGFRvIII^D^ CAR T-cells alone, EGFRvIII^D^ CAR/EGFR nsCAR T-cells showed consistently higher cytotoxicity across the tested effector-to-target ratios (Fig. 6), suggesting that the effect of nsCAR co-expression was not restricted to a single effector-to-target condition. Time-resolved cytotoxicity assays further revealed enhanced killing by EGFRvIII^D^ CAR/EGFR nsCAR T-cells at early time points compared with the corresponding CD19 nsCAR control, although these differences were not statistically significant at 6–10 h (Fig. 7). After 20 h, EGFR nsCAR co-expression resulted in significantly increased cytotoxicity across the tested effector-to-target ratios. Imaging experiments further demonstrated that both EGFRvIII CAR and EGFR nsCAR molecules co-localized at the same T-cell–tumor-cell contact interface, supporting simultaneous engagement of the two receptors. The functional consequences of this co-recruitment for parameters such as contact duration, receptor enrichment, or granule polarization remain to be determined [72]. In control experiments, nsCARs alone did not mediate cytotoxicity. Moreover, a CD19 nsCAR, which does not bind to HNSCC cells, was not recruited to the IS and did not enhance cytotoxicity of EGFRvIII^D^ CAR T-cells, supporting the conclusion that the enhancement by the nsCAR constructs depends on cooperation with a signaling-competent CAR.

The differences in the cytotoxic activity between EGFRvIII^D^ and EGFRvIII^E^ CARs likely depend on multiple parameters, including the target antigen density in combination with the specific affinity and avidity of the scFvs used [73, 74], characteristics of the hinge region within the CAR construct [75, 76], the antigen accessibility and location in the target cell membrane [77], or the epitope positioning on the extracellular domain [77]. High-affinity CARs or membrane-proximal epitopes can enhance effective receptor engagement and promote IS formation, particularly under conditions of low target antigen density [78, 79]. Notably, we also explored the broader dual-antigen concept by combining the CD44v6 CAR with the EGFR nsCAR. In this setting, EGFR nsCAR co-expression did not significantly enhance CD44v6 CAR-mediated cytotoxicity. This may partly reflect the already high baseline activity of the CD44v6 CAR or differences in antigen organization at the target-cell membrane: CD44v6 is preferentially found in lipid‑raft‑associated areas, while EGFR is organized in nanoclusters or microdomains whose signaling depends on the local lipid environment [80–82]. Here, it would be interesting to evaluate in the future whether a CD44v6 CAR construct with lower affinity/avidity can benefit from co-expression of the EGFR nsCAR, as both antigens may need to co-localize in close proximity within the same membrane region or immunological synapse for improved killing. Furthermore, evaluating alternative CAR/nsCAR combinations can be important to better define the impact of co-expressing nsCARs on the killing efficiency of CAR T-cells. Although the most pronounced benefit of nsCAR co-expression in the present study was observed in the context of dual-epitope engagement of EGFRvIII, the broader nsCAR concept may also be applicable to independently expressed tumor-associated antigens. In this context, several TAAs, which are often abundantly present on malignant cells, may represent promising targets for nsCARs when combined with TSAs like EGFRvIII in the signaling-competent CAR construct [83].

However, an important consideration for the translation of the EGFR nsCAR approach is the expression of EGFR in normal epithelial tissues. We previously demonstrated that a signaling-competent CAR containing the same cetuximab-derived scFv efficiently recognized and lysed normal human oral keratinocytes [37]. While the EGFR nsCAR used in the present study lacks intracellular signaling domains and therefore cannot independently trigger CAR-mediated cytotoxicity, binding of the T-cells with a nsCAR to physiological EGFR-positive cells remains possible and could potentially affect T-cell distribution, retention, or interactions with normal tissues.

### Additional strategies to enhance CAR T-cell cytotoxicity

Modulating signaling pathways in CAR T-cells is an interesting possibility to improve their killing activity under conditions of low target antigen expression and limited CAR T-cell persistence or functional activity. For example, the CD19 CAR T-cell killing efficiency of CD19-low tumor cells was vastly improved in vitro and in vivo by using CD38 chimeric costimulatory receptors (CCRs) that contained CD28, 4-1BB, or both as costimulatory domains, but did not induce tonic signaling due to the absence of a CD3ζ domain [84]. In addition, utilizing 4-1BB/CD137 agonists in a BiTE format, targeting either an antigen on the tumor cells or FAP in the TME, might become another promising *off-the-shelf* therapy to improve the basic cytotoxic activity of both BiTE and CAR T-cell therapies (reviewed in [85]). Finally, strengthening the IS is increasingly recognized as a powerful strategy to overcome the barriers imposed by low antigen density and the solid TME [72], e.g. by increasing LFA-1 or ICAM-1 interactions [86, 87] or by treatment with the thalidomide derivative lenalidomide that led to enhanced immunological synapses between EGFRvIII CAR T-cells and glioblastoma targets as determined by CD11a and F-actin polymerization [88]. High-quality IS formation is characterized by elevated F-actin accumulation, efficient lytic granule polarization, and robust polarization of key signaling molecules within the synapse [72]. In this context, the nsCAR approach fits within a broader framework of strategies that modulate receptor architecture and CAR–tumor interactions, while remaining conceptually simple.

A limitation of the present study is that the functional analyses were performed in two-dimensional in vitro culture systems. Thus, the current experiments do not address whether nsCAR co-expression affects CAR T-cell trafficking, tumor infiltration, or persistence within an intact solid-tumor microenvironment. Also, interactions of nsCAR T-cells with stromal cells, extracellular matrix components, and immunosuppressive cell populations such as tumor-associated macrophages and myeloid-derived suppressor cells were not analyzed. Future studies using more complex three-dimensional and in vivo tumor models need to determine whether the enhanced cytotoxicity observed in vitro translates into improved antitumor activity under these conditions.

## Conclusion

The present study provides an in vitro proof of concept that additional non-signaling receptor engagement can enhance CAR T-cell cytotoxicity in selected CAR and target contexts, with the most pronounced effect observed for the EGFRvIII^D^ CAR under intermediate target antigen expression. Further studies are required to determine whether these findings translate into improved antitumor activity, persistence, and functionality in vivo. The impact of nsCAR engagement on cytokine secretion, T-cell activation, degranulation, exhaustion, and memory formation warrants detailed studies using longitudinal functional and transcriptional profiling. In addition, the current study evaluated a limited panel of CAR architectures and tumor models, and broader validation of different CAR designs and tumor target antigens is necessary to determine the generalizability of nsCAR strategies across diverse antigen landscapes. Here, deeper mechanistic investigations using quantitative imaging approaches, including F-actin accumulation assays, granule-polarization metrics, and high-resolution synapse biophysics [72, 89, 90], will help determine whether receptor co-recruitment is associated with specific structural or functional changes at the T-cell–target-cell interface.

## Supplementary Information

Supplementary Material 1.

Supplementary Material 2.

## Data Availability

The datasets used and/or analysed during the current study are available from the corresponding author on reasonable request.

## References

[1] Sun H, et al. Global burden of head and neck cancer: Epidemiological transitions, inequities, and projections to 2050. Front Oncol. 2025;15:1665019. 10.3389/fonc.2025.1665019 PMC12507627 41079071

[2] Barsouk A et al. Epidemiology, Risk Factors, and Prevention of Head and Neck Squamous Cell Carcinoma. Med Sci (Basel). 2023;11(2):42. 10.3390/medsci11020042 PMC10304137 37367741

[3] Machiels JP, et al. Squamous cell carcinoma of the oral cavity, larynx, oropharynx and hypopharynx: EHNS-ESMO-ESTRO Clinical Practice Guidelines for diagnosis, treatment and follow-up. Ann Oncol. 2020;31(11):1462–75. 10.1016/j.annonc.2020.07.011 33239190

[4] Borcoman E, Marret G, Le Tourneau C. Paradigm change in first-line treatment of recurrent and/or metastatic Head and Neck Squamous Cell Carcinoma. Cancers (Basel). 2021;13(11). 10.3390/cancers13112573 PMC8197177 34073885

[5] Ruffin AT, et al. Improving head and neck cancer therapies by immunomodulation of the tumour microenvironment. Nat Rev Cancer. 2023;23(3):173–88. 10.1038/s41568-022-00531-9 PMC9992112 36456755

[6] June CH, Sadelain M. Chimeric Antigen Receptor Therapy. N Engl J Med. 2018;379(1):64–73. 10.1056/NEJMra1706169 PMC7433347 29972754

[7] Uslu U, June CH. Beyond the blood: expanding CAR T cell therapy to solid tumors. Nat Biotechnol. 2025;43(4):506–15. 10.1038/s41587-024-02446-2 39533105

[8] Locatelli F, et al. GD2-targeting CAR T cells in high-risk neuroblastoma: a phase 1/2 trial. Nat Med. 2025;31(11):3689–3699. 10.1038/s41591-025-03874-6 40841488

[9] Steffin D, et al. Interleukin-15-armoured GPC3 CAR T cells for patients with solid cancers. Nature. 2025;637(8047):940–6. 10.1038/s41586-024-08261-8 PMC12704925 39604730

[10] Guo J, et al. Challenges and breakthroughs: current landscape and future prospects of CAR-T cell therapy clinical trials for solid tumors. Front Oncol. 2025;15:1652329. 10.3389/fonc.2025.1652329 PMC12440771 40969260

[11] Arteaga CL, Engelman JA. ERBB receptors: from oncogene discovery to basic science to mechanism-based cancer therapeutics. Cancer Cell. 2014;25(3):282–303. 10.1016/j.ccr.2014.02.025 PMC4018830 24651011

[12] Yao Y, et al. Clinical utility of PDX cohorts to reveal biomarkers of intrinsic resistance and clonal architecture changes underlying acquired resistance to cetuximab in HNSCC. Signal Transduct Target Ther. 2022;7(1):73. 10.1038/s41392-022-00908-0 PMC8904860 35260570

[13] Huang HS, et al. The enhanced tumorigenic activity of a mutant epidermal growth factor receptor common in human cancers is mediated by threshold levels of constitutive tyrosine phosphorylation and unattenuated signaling. J Biol Chem. 1997;272(5):2927–35. 10.1074/jbc.272.5.2927 9006938

[14] Guo G, et al. Ligand-Independent EGFR Signaling. Cancer Res. 2015;75(17):3436–41. 10.1158/0008-5472.CAN-15-0989 PMC4558210 26282175

[15] Wikstrand CJ, et al. Monoclonal antibodies against EGFRvIII are tumor specific and react with breast and lung carcinomas and malignant gliomas. Cancer Res. 1995;55(14):3140–8. 7606735

[16] Voldborg BR, et al. Epidermal growth factor receptor (EGFR) and EGFR mutations, function and possible role in clinical trials. Ann Oncol. 1997;8(12):1197–206. 10.1023/a:1008209720526 9496384

[17] Moscatello DK, et al. Transformational and altered signal transduction by a naturally occurring mutant EGF receptor. Oncogene. 1996;13(1):85–96. 8700557

[18] Batra SK, et al. Epidermal growth factor ligand-independent, unregulated, cell-transforming potential of a naturally occurring human mutant EGFRvIII gene. Cell Growth Differ. 1995;6(10):1251–9. 8845302

[19] Katanasaka Y, et al. Epidermal growth factor receptor variant type III markedly accelerates angiogenesis and tumor growth via inducing c-myc mediated angiopoietin-like 4 expression in malignant glioma. Mol Cancer. 2013;12:31. 10.1186/1476-4598-12-31 PMC3641008 23617883

[20] Bonavia R, et al. EGFRvIII promotes glioma angiogenesis and growth through the NF-kappaB, interleukin-8 pathway. Oncogene. 2012;31(36):4054–66. 10.1038/onc.2011.563 PMC3537826 22139077

[21] Feng H, et al. EGFRvIII stimulates glioma growth and invasion through PKA-dependent serine phosphorylation of Dock180. Oncogene. 2014;33(19):2504–12. 10.1038/onc.2013.198 PMC3883905 23728337

[22] O’Rourke DM et al. A single dose of peripherally infused EGFRvIII-directed CAR T cells mediates antigen loss and induces adaptive resistance in patients with recurrent glioblastoma. Sci Transl Med. 2017;9(399):eaaa0984. 10.1126/scitranslmed.aaa0984 PMC5762203 28724573

[23] Goff SL, et al. Pilot Trial of Adoptive Transfer of Chimeric Antigen Receptor-transduced T Cells Targeting EGFRvIII in Patients With Glioblastoma. J Immunother. 2019;42(4):126–35. 10.1097/CJI.0000000000000260 PMC6691897 30882547

[24] Choi BD, et al. Intraventricular CARv3-TEAM-E T Cells in Recurrent Glioblastoma. N Engl J Med. 2024;390(14):1290–8. 10.1056/NEJMoa2314390 PMC11162836 38477966

[25] Choi BD, et al. CAR-T cells secreting BiTEs circumvent antigen escape without detectable toxicity. Nat Biotechnol. 2019;37(9):1049–58. 10.1038/s41587-019-0192-1 31332324

[26] Ramakrishna S, et al. Modulation of Target Antigen Density Improves CAR T-cell Functionality and Persistence. Clin Cancer Res. 2019;25(17):5329–41. 10.1158/1078-0432.CCR-18-3784 PMC8290499 31110075

[27] Walker AJ, et al. Tumor Antigen and Receptor Densities Regulate Efficacy of a Chimeric Antigen Receptor Targeting Anaplastic Lymphoma Kinase. Mol Ther. 2017;25(9):2189–201. 10.1016/j.ymthe.2017.06.008 PMC5589087 28676342

[28] Fry TJ, et al. CD22-targeted CAR T cells induce remission in B-ALL that is naive or resistant to CD19-targeted CAR immunotherapy. Nat Med. 2018;24(1):20–8. 10.1038/nm.4441 PMC5774642 29155426

[29] Watanabe K, et al. Target antigen density governs the efficacy of anti-CD20-CD28-CD3 zeta chimeric antigen receptor-modified effector CD8 + T cells. J Immunol. 2015;194(3):911–20. 10.4049/jimmunol.1402346 25520398

[30] Majzner RG, et al. Tuning the Antigen Density Requirement for CAR T-cell Activity. Cancer Discov. 2020;10(5):702–23. 10.1158/2159-8290.CD-19-0945 PMC7939454 32193224

[31] Barisa M, et al. Functional avidity of anti-B7H3 CAR-T constructs predicts antigen density thresholds for triggering effector function. Nat Commun. 2025;16(1):7196. 10.1038/s41467-025-61427-4 PMC12325614 40764385

[32] Schneider D, et al. A tandem CD19/CD20 CAR lentiviral vector drives on-target and off-target antigen modulation in leukemia cell lines. J Immunother Cancer. 2017;5:42. 10.1186/s40425-017-0246-1 PMC5433150 28515942

[33] Ruella M, et al. Dual CD19 and CD123 targeting prevents antigen-loss relapses after CD19-directed immunotherapies. J Clin Invest. 2016;126(10):3814–26. 10.1172/JCI87366 PMC5096828 27571406

[34] Hegde M, et al. Combinational targeting offsets antigen escape and enhances effector functions of adoptively transferred T cells in glioblastoma. Mol Ther. 2013;21(11):2087–101. 10.1038/mt.2013.185 PMC3831041 23939024

[35] Fernandez de Larrea C, et al. Defining an Optimal Dual-Targeted CAR T-cell Therapy Approach Simultaneously Targeting BCMA and GPRC5D to Prevent BCMA Escape-Driven Relapse in Multiple Myeloma. Blood Cancer Discov. 2020;1(2):146–54. 10.1158/2643-3230.BCD-20-0020 PMC7575057 33089218

[36] Virts EL, et al. AluY-mediated germline deletion, duplication and somatic stem cell reversion in UBE2T defines a new subtype of Fanconi anemia. Hum Mol Genet. 2015;24(18):5093–108. 10.1093/hmg/ddv227 PMC4550815 26085575

[37] Haist C, et al. Engineering a single-chain variable fragment of cetuximab for CAR T-cell therapy against head and neck squamous cell carcinomas. Oral Oncol. 2022;129:105867. 10.1016/j.oraloncology.2022.105867 35468475

[38] Sok JC, et al. Mutant epidermal growth factor receptor (EGFRvIII) contributes to head and neck cancer growth and resistance to EGFR targeting. Clin Cancer Res. 2006;12(17):5064–73. 10.1158/1078-0432.CCR-06-0913 16951222

[39] Nakano M, et al. CYP4V2 in Bietti’s crystalline dystrophy: ocular localization, metabolism of omega-3-polyunsaturated fatty acids, and functional deficit of the p.H331P variant. Mol Pharmacol. 2012;82(4):679–86. 10.1124/mol.112.080085 PMC3463217 22772592

[40] Yu X, et al. Lentiviral vectors with two independent internal promoters transfer high-level expression of multiple transgenes to human hematopoietic stem-progenitor cells. Mol Ther. 2003;7(6):827–38. 10.1016/s1525-0016(03)00104-7 12788657

[41] Roellecke K, et al. Optimized human CYP4B1 in combination with the alkylator prodrug 4-ipomeanol serves as a novel suicide gene system for adoptive T-cell therapies. Gene Ther. 2016;23(7):615–26. 10.1038/gt.2016.38 27092941

[42] Haist C, et al. CD44v6-targeted CAR T-cells specifically eliminate CD44 isoform 6 expressing head/neck squamous cell carcinoma cells. Oral Oncol. 2021;116:105259. 10.1016/j.oraloncology.2021.105259 33895463

[43] Qin JY, et al. Systematic comparison of constitutive promoters and the doxycycline-inducible promoter. PLoS ONE. 2010;5(5):e10611. 10.1371/journal.pone.0010611 PMC2868906 20485554

[44] Greulich H, et al. Functional analysis of receptor tyrosine kinase mutations in lung cancer identifies oncogenic extracellular domain mutations of ERBB2. Proc Natl Acad Sci U S A. 2012;109(36):14476–81. 10.1073/pnas.1203201109 PMC3437859 22908275

[45] Johnson LA, et al. Rational development and characterization of humanized anti-EGFR variant III chimeric antigen receptor T cells for glioblastoma. Sci Transl Med. 2015;7(275):275ra22. 10.1126/scitranslmed.aaa4963 PMC4467166 25696001

[46] Morgan RA, et al. Recognition of glioma stem cells by genetically modified T cells targeting EGFRvIII and development of adoptive cell therapy for glioma. Hum Gene Ther. 2012;23(10):1043–53. 10.1089/hum.2012.041 PMC3472555 22780919

[47] Bister A, et al. A novel CD34-derived hinge for rapid and efficient detection and enrichment of CAR T cells. Mol Ther Oncolytics. 2021;23:534–46. 10.1016/j.omto.2021.11.003 PMC8640169 34901395

[48] Rossig C, et al. Adoptive cellular immunotherapy with CD19-specific T cells. Klin Padiatr. 2005;217(6):351–6. 10.1055/s-2005-872521 16307422

[49] Bister A, et al. Optimized NGFR-derived hinges for rapid and efficient enrichment and detection of CAR T cells in vitro and in vivo. Mol Ther Oncolytics. 2022;26:120–34. 10.1016/j.omto.2022.05.012 PMC9240717 35795096

[50] Soldierer M, et al. Genetic Engineering and Enrichment of Human NK Cells for CAR-Enhanced Immunotherapy of Hematological Malignancies. Front Immunol. 2022;13:847008. 10.3389/fimmu.2022.847008 PMC9022481 35464442

[51] Del Vecchio CA, et al. EGFRvIII gene rearrangement is an early event in glioblastoma tumorigenesis and expression defines a hierarchy modulated by epigenetic mechanisms. Oncogene. 2013;32(21):2670–81. 10.1038/onc.2012.280 22797070

[52] An Z, et al. EGFR and EGFRvIII coopt host defense pathways promoting progression in glioblastoma. Neuro Oncol. 2025;27(2):383–97. 10.1093/neuonc/noae182 PMC11812036 39248287

[53] Gan HK, Kaye AH, Luwor RB. The EGFRvIII variant in glioblastoma multiforme. J Clin Neurosci. 2009;16(6):748–54. 10.1016/j.jocn.2008.12.005 19324552

[54] Makabe K, et al. Anti-EGFR antibody 528 binds to domain III of EGFR at a site shifted from the cetuximab epitope. Sci Rep. 2021;11(1):5790. 10.1038/s41598-021-84171-3 PMC7952593 33707468

[55] Fleischer LC, et al. Non-signaling Chimeric Antigen Receptors Enhance Antigen-Directed Killing by gammadelta T Cells in Contrast to alphabeta T Cells. Mol Ther Oncolytics. 2020;18:149–60. 10.1016/j.omto.2020.06.003 PMC7341062 32671190

[56] Pollok KE, et al. High-efficiency gene transfer into normal and adenosine deaminase-deficient T lymphocytes is mediated by transduction on recombinant fibronectin fragments. J Virol. 1998;72(6):4882–92. 10.1128/jvi.72.6.4882-4892.1998 PMC110042 9573255

[57] Lamplugh ZL, et al. Microenvironmental regulation of solid tumour resistance to CAR T cell therapy. Nat Rev Immunol. 2026;26(3):230–48. 10.1038/s41577-025-01229-3 41087553

[58] Wheeler SE, et al. Epidermal growth factor receptor variant III mediates head and neck cancer cell invasion via STAT3 activation. Oncogene. 2010;29(37):5135–45. 10.1038/onc.2009.279 PMC2940981 20622897

[59] Zhang HD, et al. [Preliminary study on the expression of EGFRvⅢ and CXCR4 in head and neck squamous cell carcinoma]. Lin Chuang Er Bi Yan Hou Tou Jing Wai Ke Za Zhi. 2017;31(11):817–21. 10.13201/j.issn.1001-1781.2017.11.001 29774993

[60] Melchers LJ, et al. Head and neck squamous cell carcinomas do not express EGFRvIII. Int J Radiat Oncol Biol Phys. 2014;90(2):454–62. 10.1016/j.ijrobp.2014.06.035 25304797

[61] Thomas Koch D, et al. Epidermal growth factor receptor variant III in head and neck squamous cell carcinoma is not relevant for targeted therapy and irradiation. Oncotarget. 2017;8(20):32668–82. 10.18632/oncotarget.15949 PMC5464818 28427242

[62] Khattri A, et al. Rare occurrence of EGFRvIII deletion in head and neck squamous cell carcinoma. Oral Oncol. 2015;51(1):53–8. 10.1016/j.oraloncology.2014.08.014 25255959

[63] Stec WJ, et al. Cell line with endogenous EGFRvIII expression is a suitable model for research and drug development purposes. Oncotarget. 2016;7(22):31907–25. 10.18632/oncotarget.8201 PMC5077985 27004406

[64] Gedeon PC, et al. Rindopepimut: anti-EGFRvIII peptide vaccine, oncolytic. Drugs Future. 2013;38(3):147–55. 10.1358/dof.2013.038.03.1933992 PMC4317793 25663738

[65] Weller M, et al. Rindopepimut with temozolomide for patients with newly diagnosed, EGFRvIII-expressing glioblastoma (ACT IV): a randomised, double-blind, international phase 3 trial. Lancet Oncol. 2017;18(10):1373–85. 10.1016/S1470-2045(17)30517-X 28844499

[66] Lassman AB, et al. Depatuxizumab mafodotin in EGFR-amplified newly diagnosed glioblastoma: A phase III randomized clinical trial. Neuro Oncol. 2023;25(2):339–50. 10.1093/neuonc/noac173 PMC9925712 35849035

[67] van den Bent M, et al. Efficacy of depatuxizumab mafodotin (ABT-414) monotherapy in patients with EGFR-amplified, recurrent glioblastoma: results from a multi-center, international study. Cancer Chemother Pharmacol. 2017;80(6):1209–17. 10.1007/s00280-017-3451-1 PMC5686264 29075855

[68] Zhu H, et al. EGFRvIII-CAR-T Cells with PD-1 Knockout Have Improved Anti-Glioma Activity. Pathol Oncol Res. 2020;26(4):2135–41. 10.1007/s12253-019-00759-1 31989402

[69] Miao H, et al. EGFRvIII-specific chimeric antigen receptor T cells migrate to and kill tumor deposits infiltrating the brain parenchyma in an invasive xenograft model of glioblastoma. PLoS ONE. 2014;9(4):e94281. 10.1371/journal.pone.0094281 PMC3983153 24722266

[70] Li J, et al. Optimizing CAR T cell therapy for solid tumours: a clinical perspective. Nat Rev Clin Oncol. 2025;22(12):953–68. 10.1038/s41571-025-01075-1 41039013

[71] Tu Z, et al. Barriers and solutions for CAR-T therapy in solid tumors. Cancer Gene Ther. 2025;32(9):923–34. 10.1038/s41417-025-00931-7 40579466

[72] Liu D, et al. The Role of Immunological Synapse in Predicting the Efficacy of Chimeric Antigen Receptor (CAR) Immunotherapy. Cell Commun Signal. 2020;18(1):134. 10.1186/s12964-020-00617-7 PMC7446110 32843053

[73] Greenman R, et al. Shaping Functional Avidity of CAR T Cells: Affinity, Avidity, and Antigen Density That Regulate Response. Mol Cancer Ther. 2021;20(5):872–84. 10.1158/1535-7163.MCT-19-1109 33649106

[74] Mao R, Kong W, He Y. The affinity of antigen-binding domain on the antitumor efficacy of CAR T cells: Moderate is better. Front Immunol. 2022;13:1032403. 10.3389/fimmu.2022.1032403 PMC9618871 36325345

[75] Guest RD, et al. The role of extracellular spacer regions in the optimal design of chimeric immune receptors: evaluation of four different scFvs and antigens. J Immunother. 2005;28(3):203–11. 10.1097/01.cji.0000161397.96582.59 15838376

[76] Hudecek M, et al. Receptor affinity and extracellular domain modifications affect tumor recognition by ROR1-specific chimeric antigen receptor T cells. Clin Cancer Res. 2013;19(12):3153–64. 10.1158/1078-0432.CCR-13-0330 PMC3804130 23620405

[77] Hanssens H, et al. The antigen-binding moiety in the driver’s seat of CARs. Med Res Rev. 2022;42(1):306–42. 10.1002/med.21818 PMC9292017 34028069

[78] Xiong Y, Libby KA, Su X. The physical landscape of CAR-T synapse. Biophys J. 2024;123(15):2199–210. 10.1016/j.bpj.2023.09.004 PMC11331049 37715447

[79] Harrer DC, et al. Fine-tuning the antigen sensitivity of CAR T cells: emerging strategies and current challenges. Front Immunol. 2023;14:1321596. 10.3389/fimmu.2023.1321596 PMC10711209 38090558

[80] Balbis A, Posner BI. Compartmentalization of EGFR in cellular membranes: role of membrane rafts. J Cell Biochem. 2010;109(6):1103–8. 10.1002/jcb.22505 20143338

[81] Ghatak S, et al. Chemotherapy induces feedback up-regulation of CD44v6 in colorectal cancer initiating cells through beta-catenin/MDR1 signaling to sustain chemoresistance. Front Oncol. 2022;12:906260. 10.3389/fonc.2022.906260 PMC9623568 36330477

[82] Oliferenko S, et al. Analysis of CD44-containing lipid rafts: Recruitment of annexin II and stabilization by the actin cytoskeleton. J Cell Biol. 1999;146(4):843–54. 10.1083/jcb.146.4.843 PMC2156143 10459018

[83] von Witzleben A et al. HNSCC: Tumour Antigens and Their Targeting by Immunotherapy. Cells. 2020;9(9):2103. 10.3390/cells9092103 PMC7564543 32942747

[84] Katsarou A, et al. Combining a CAR and a chimeric costimulatory receptor enhances T cell sensitivity to low antigen density and promotes persistence. Sci Transl Med. 2021;13(623):eabh1962. 10.1126/scitranslmed.abh1962 PMC9869449 34878825

[85] Claus C, Ferrara-Koller C, Klein C. The emerging landscape of novel 4-1BB (CD137) agonistic drugs for cancer immunotherapy. MAbs. 2023;15(1):2167189. 10.1080/19420862.2023.2167189 PMC9897756 36727218

[86] Gao Y et al. Optimization of T Cell Redirecting Strategies: Obtaining Inspirations From Natural Process of T Cell Activation. Front Immunol. 2021;12:664329. 10.3389/fimmu.2021.664329 PMC8107274 33981310

[87] Espie D, Donnadieu E. New insights into CAR T cell-mediated killing of tumor cells. Front Immunol. 2022;13:1016208. 10.3389/fimmu.2022.1016208 PMC9521365 36189315

[88] Kuramitsu S, et al. Lenalidomide enhances the function of chimeric antigen receptor T cells against the epidermal growth factor receptor variant III by enhancing immune synapses. Cancer Gene Ther. 2015;22(10):487–95. 10.1038/cgt.2015.47 26450624

[89] Banerjee PP, Orange JS. Quantitative measurement of F-actin accumulation at the NK cell immunological synapse. J Immunol Methods. 2010;355(1–2):1–13. 10.1016/j.jim.2010.02.003 PMC2854315 20171970

[90] Jang JH, et al. Imaging of Cell-Cell Communication in a Vertical Orientation Reveals High-Resolution Structure of Immunological Synapse and Novel PD-1 Dynamics. J Immunol. 2015;195(3):1320–30. 10.4049/jimmunol.1403143 PMC4506859 26123352

