# Supplementary material for: Co-expression of non-signaling CARs enhances EGFRvIII-specific CAR T-cell cytotoxicity against head and neck squamous cell carcinoma
Source: BMC Cancer. 2026 Sep 29;26:1149. doi: 10.1186/s12885-026-17058-z (PMC13625461; doi:10.1186/s12885-026-17058-z)
Supplement: Supplementary file 1 [file 12885_2026_17058_MOESM1_ESM.docx]

**Figure S1: Comparison of internal promoter activity based on GFP expression in a SIN lentiviral vector. *(A) Schematic representation of the LV expression vector.*** *The internal promoters drive the expression levels of the GFP transgene.* ***(B) Evaluation of infectious titer (TU/mL) and GFP mean fluorescence intensity (MFI) of LV vectors in EGFR KO cell lines UM-14C^KO^ and UT-24A^KO^.*** *Cells were analyzed for GFP expression 5 days after transduction by flow cytometry. Only samples containing fewer than 25% GFP-positive cells were included in the flow cytometry analysis. Means ± SEM from three independent experiments are shown.*

***Figure S2: Co-expression of CAR and nsCAR constructs. (A) Enrichment of T-cells co-expressing CAR and nsCAR constructs.*** *Dot plots show CD34 expression (x-axis) versus NGFR expression (y-axis). Primary human T-cells expressing only the EGFR nsCAR were enriched with NGFR microbeads, whereas cells expressing only the CD44v6 CAR or the CD44v6 CAR together with the EGFR nsCAR were enriched using CD34 microbeads. Before and after MACS separation, gene transfer efficiency was assessed by staining with FITC-conjugated CD34 and APC-conjugated NGFR antibodies. Representative experiments are shown. Numbers indicate the percentages of cells within each quadrant.* ***(B) Representative flow-cytometric histograms showing surface expression of the signaling CAR after MACS enrichment.*** *CAR expression was assessed by CD34-FITC staining in T-cells expressing the indicated CAR alone (blue) or co-expressing the CAR with either the EGFR nsCAR (red) or the CD19 nsCAR (orange). Histograms were normalized to mode and are shown for EGFRvIII^D^, EGFRvIII^E^, CD33, and CD44v6 CAR T-cells.*
